# Spatiotemporal coordination of the RSF1-PLK1-Aurora B cascade establishes mitotic signaling platforms

**DOI:** 10.1038/s41467-021-26220-z

**Published:** 2021-10-11

**Authors:** Ho-Soo Lee, Sunwoo Min, Ye-Eun Jung, Sunyoung Chae, June Heo, Jae-Ho Lee, TaeSoo Kim, Ho-Chul Kang, Makoto Nakanish, Sun-Shin Cha, Hyeseong Cho

**Affiliations:** 1 Department of Biochemistry and Molecular Biology, Ajou University School of Medicine, Suwon, 16499 Korea; 2 Department of Chemistry and Nanoscience, Ewha Womans University, Seoul, 03760 Republic of Korea; 3 Institute of Medical Science, Ajou University School of Medicine, Suwon, 16499 Korea; 4 Department of Biomedical Sciences, Graduate School of Ajou University, Suwon, 16499 Korea; 5 Department of Life Science, Ewha Womans University, Seoul, 03760 Korea; 6 Department of Physiology, Ajou University School of Medicine, Suwon, Korea; 7 Division of Cancer Cell Biology, The University of Tokyo, Tokyo, 108-8639 Japan

**Keywords:** Chromosome segregation, Kinetochores

## Abstract

The chromatin remodeler RSF1 enriched at mitotic centromeres is essential for proper chromosome alignment and segregation and underlying mechanisms remain to be disclosed. We here show that PLK1 recruitment by RSF1 at centromeres creates an activating phosphorylation on Thr236 in the activation loop of Aurora B and this is indispensable for the Aurora B activation. In structural modeling the phosphorylated Thr236 enhances the base catalysis by Asp200 nearby, facilitating the Thr232 autophosphorylation. Accordingly, RSF1-PLK1 is central for Aurora B-mediated microtubule destabilization in error correction. However, under full microtubule-kinetochore attachment RSF1-PLK1 positions at kinetochores, halts activating Aurora B and phosphorylates BubR1, regardless of tension. Spatial movement of RSF1-PLK1 to kinetochores is triggered by Aurora B-mediated phosphorylation of centromeric histone H3 on Ser28. We propose a regulatory RSF1-PLK1 axis that spatiotemporally controls on/off switch on Aurora B. This feedback circuit among RSF1-PLK1-Aurora B may coordinate dynamic microtubule-kinetochore attachment in early mitosis when full tension yet to be generated.

## Abstract (web summary)

During cell division, chromosome alignment is engendered by connection of microtubules to kinetochores, coordinated by Aurora B and PLK1. Here, the authors show that the RSF1-PLK1 axis creates an activating phosphorylation on T236 in the GT motif of Aurora B and this is indispensable for Aurora B activation.

## Introduction

A key mission in mitosis is accurate chromosome segregation, which requires exquisite coordination of several mitotic kinases and phosphatases^1–3^. Chromosome alignment is engendered by connection of spindle microtubules (MTs) emanating from opposite spindle poles to the macromolecular complex of kinetochores (KTs) assembled on centromeric nucleosomes^4,5^. A single unattached chromosome activates the spindle assembly checkpoint (SAC), delaying the anaphase onset^6–8^. The regulated phosphorylation of proteins by Aurora B kinase and polo-like kinase 1 (PLK1) is important for the maintenance of MT–KT attachment and error correction, as well as activation of the SAC^9–15^. Aurora B kinase corrects erroneous MT–KT attachment leading to sister-KT bi-orientation^13^. This is driven by the Aurora B-mediated phosphorylation of the Ndc80 complex proteins that triggers a decrease in the MT-binding affinity to the KT^16^. On the other hand, PLK1 stabilizes MT–KT attachment through creating a binding site for protein phosphatase PP2A on BubR1 through phosphorylation^17–19^. Recruitment of PP2A at KTs counteracts Aurora B kinase activity^20^. This elegant and coordinated system at centromeres and KTs allows cells to perfect timing for correct chromosome segregation. At centromeres, PLK1 phosphorylates Borealin and Survivin in the chromosome passenger complex (CPC) and this is important for Aurora B docking and its activation as well^21,22^. Therefore, PLK1 and Aurora B are highly intercorrelated but are functionally antagonistic for MT–KT attachment^23^. Thus, it still raises the question how the functional compromise between Aurora B and PLK1 is achieved for timely chromosome segregation in these intercorrelated circuits.

The fundamental action of chromatin remodeling complexes in cells is to remove the nucleosome barrier during transcription, DNA replication, and repair^24,25^. Remodeling and spacing factor (RSF) is composed of SNF2H ATPase and RSF1 as an accessory subunit^26,27^. The RSF complex is shown to facilitate transcription initiation for the chromatin template^27^. Interestingly, the RSF complex was co-purified with CENP-A nucleosomes along with constitutive centromere-associated network (CCAN) components. Accordingly, RSF1 was suggested to be involved in the assembly of centromeric chromatin and stabilization of the CENP-A complex^28,29^. In addition, RSF1 contributes to DNA double-strand break repair and one of the ways to promote DNA repair is to recruit the centromeric proteins CENP-S and CENP-X to the DNA lesion^30–32^. We recently reported that localization of RSF1 is confined to centromeres in early mitosis. RSF1 is an essential component for proper chromosome alignment and segregation, because loss of RSF1 leads to severe defects in chromosome alignment and segregation. This is at least partly caused by lack of PLK1 accumulation at centromeric regions. PLK1 recruited by RSF1 stabilizes MT–KT attachment and proper chromosome alignment^33^. In addition, RSF1 interaction with histone deacetylase 1 leads to histone H2A deacetylation and subsequent Bub1-mediated phosphorylation of H2A for proper Sgo1 deposition^34^. Thus, mitotic regulation of RSF1 has just emerged and other mitotic roles have yet to be explored.

In the present study, we provide key insights on the centromeric signaling network. The RSF1-PLK1 axis spatiotemporally controls Aurora B kinase activity in early mitosis. In RSF1-depleted cells, Aurora B kinase activity was severely damaged and this was restored by overexpression of functional PLK1. We identified a novel activating phosphorylation site Thr236 in the activation loop of Aurora B as a direct target of PLK1. Our study also reveals that the RSF1-PLK1 axis generates discrete mitotic signaling by preferentially phosphorylating Aurora B or BubR1. This phosphorylation switch may contribute to timely MT destabilization/stabilization signals.

## Results

### RSF1 regulates Aurora B kinase activity at mitotic centromeres

**Fig. 1:**
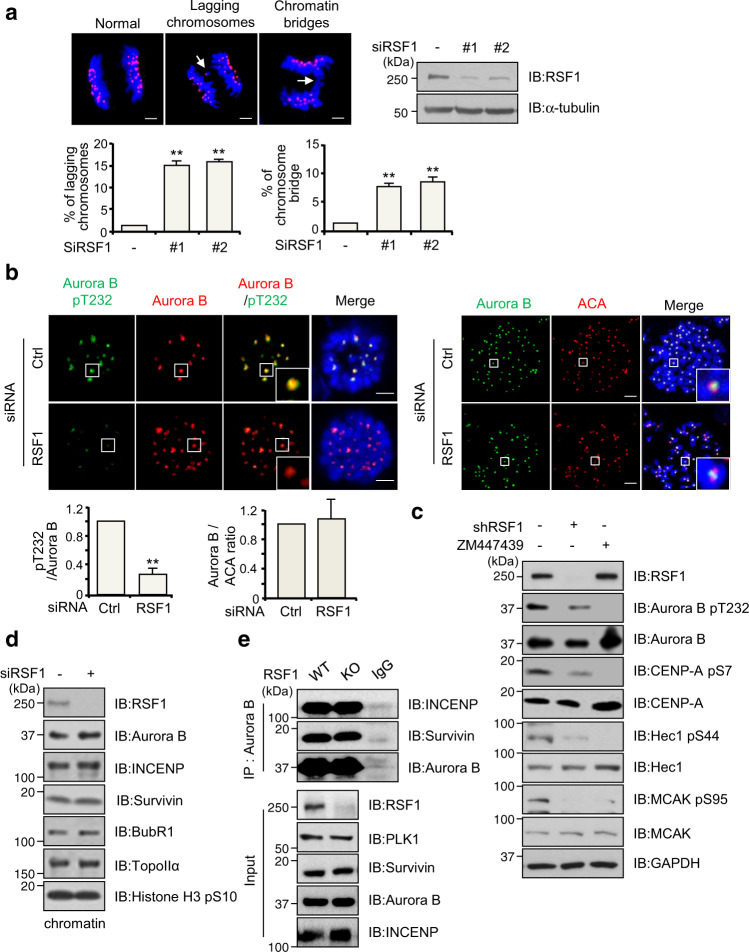
RSF1 is necessary for the Aurora B kinase activity. **a** Percentage of anaphases from RSF1 siRNA-expressing cells that display anaphase bridges or lagging chromosomes. These cells arrested at anaphase were stained with DAPI (blue) and ACA (red). Bars represent mean ± SEM from three independent experiments; siCtrl *n* = 252, siRSF1#1 *n* = 268, siRSF1#2 *n* = 172 cells. **p < 0.005 vs. control siRNA by two-sided unpaired Student’s *t*-test. Scale bar, 5 μm. **b** HeLa cells were transfected with RSF1 siRNA. Floating mitotic cells were obtained after nocodazole treatment for 4 h and subjected to immunofluorescence stained with the indicated antibodies. Nucleus was stained with DAPI (blue). The graphs represent mean ± SEM from three independent experiments showing relative signal intensity of Aurora B-pT232 (siCtrl *n* = 42 and siRSF1 *n* = 57) or Aurora B (siCtrl = 25 and siRSF1 *n* = 34) at the kinetochores. **p < 0.005 vs. control siRNA by two-sided unpaired Student’s *t*-test. Scale bar, 5 μm. **c** The HeLa cell lysates were obtained from stably expressing the control or RSF1 shRNA and control shRNA cells treated with ZM447439, Aurora B inhibitor, was treated with nocodazole for 16 h. The mitotic cell lysates were subjected to immunoblotting with indicated antibodies. **d** HeLa cells transfected with RSF1 siRNA were treated with nocodazole for 16 h. Chromatin-bound fraction was subjected to immunoblotting with the indicated antibodies. **e** Mitotic RSF1 WT or RSF1 KO HeLa cell lysates were immunoprecipitated with anti-Aurora B antibody (endogenous) or mouse IgG antibody and followed by immunoblotting with indicated antibodies. Data of **c**–**e** are representative of three independent experiments. Source data are provided as a Source Data file.

We have recently reported that RSF1 depletion resulted in severe defects in early mitotic events such as chromosome alignment at metaphase (Supplementary Fig. 1a) and sister chromatid segregation^33,34^. In RSF1-defective cells, we also noticed a considerable increase in lagging chromosomes (>15%) and chromosome bridges (~8%) (Fig. 1a). These results suggested that the error correction activity of Aurora B might be defective in these cells, because erroneous MT–KT attachment is known to lead to lagging chromosomes. We investigated the localization of the Aurora B protein and its kinase activity at centromeres using immunostaining. The localization of Aurora B at centromeres was not altered in RSF1-depleted cells arrested in prometaphase after nocodazole treatment (Fig. 1b, right panel). Notably, the phosphorylation level of Aurora B at Thr232, the autophosphorylation site essential for full activation of Aurora B^35^, was significantly reduced in RSF1-depleted cells (Fig. 1b, left panel). Consistent with the immunostaining experiments of Fig. 1b, immunoblot analysis verified that the pThr232 level of Aurora B was dramatically decreased in RSF1-depleted mitotic cell lysates, whereas Aurora B protein levels remained constant (Fig. 1c). Accordingly, the Aurora B-mediated phosphorylation of CENP-A on Ser7 was significantly reduced. Treatment of ZM447439, a potent Aurora A/B kinase inhibitor, also completely abolished the phosphorylated Aurora B and CENP-A levels. Correction of erroneous MT–KT attachments by Aurora B kinase largely relies on increased phosphorylation and activation of key KT proteins, including the MT-binding KNL1/MIS12/NDC80 complex network^36^. We found that the Aurora B-dependent phosphorylation of Hec1 (at Ser44) and MCAK (at Ser95) was also severely damaged in the shRSF1 HeLa cell line stably expressing short hairpin RNA (shRNA) targeting RSF1. In fact, the Aurora B-dependent phosphorylation of Hec1 and MCAK in RSF1-depleted cells was as low as in cells treated with ZM447439 (Fig. 1c). Thus, these data clearly demonstrate that Aurora B kinase activity is disrupted in RSF1-deficient cells and the observed aberrant mitotic phenotypes are highly likely derived from defective Aurora B function in these cells. Aurora B is a component of the CPC, containing inner centromere protein (INCENP), Survivin, and Borealin. Localization of Aurora B to the centromeres and its kinase activity rely on the assembly and multiple phosphorylation of CPC components. Analysis of the chromatin-bound fractions in these cells revealed that INCENP and Survivin levels were not altered by RSF1 depletion (Fig. 1d and Supplementary Fig. 1b, c). Likewise, co-immunoprecipitation experiments indicated that association of Aurora B with INCENP and Survivin was not changed by RSF1 depletion (Fig. 1e). These findings were consistent with our previous immunofluorescence staining data^33^. Together, our data elucidate that RSF1 regulates Aurora B kinase activity at mitotic centromeres without disturbing the CPC components.

### RSF1-PLK1 axis is required for Aurora B kinase activity

**Fig. 2:**
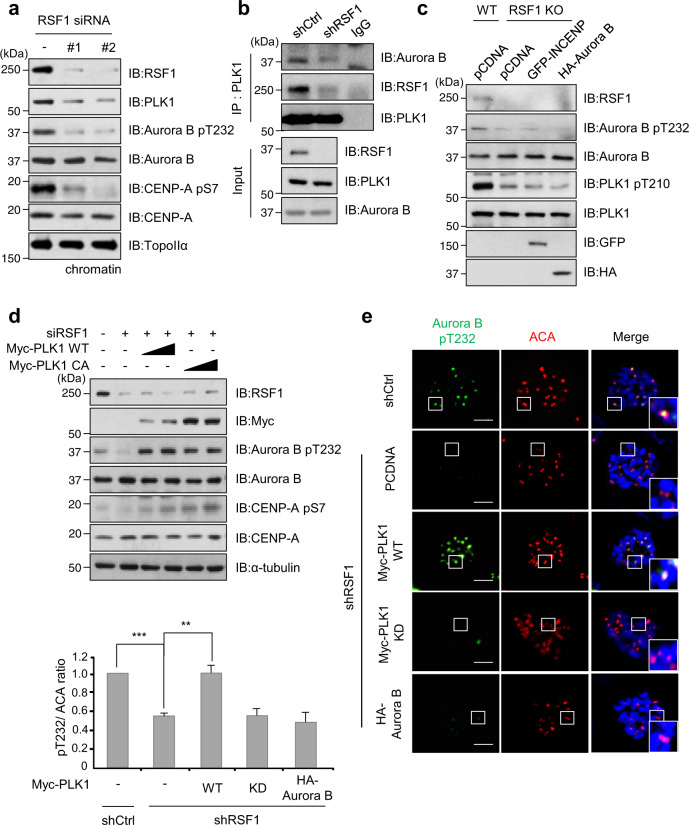
RSF1-dependent PLK1 deposition is required for the Aurora B kinase activity. **a** The HeLa cell lysates were obtained from mitotic shake-off after treatment with nocodazole for 16 h in RSF1 siRNA-expressing cells. Western blot analysis of chromatin fraction was immunoblotted for indicated antibodies. TopoII-α was used as a marker for chromatin fraction. **b** Control or RSF1 shRNA mitotic cell lysates were immunoprecipitated with anti-PLK1 antibody and immunoblotted with indicated antibodies. **c** GFP-INCENP or HA-Aurora B plasmid was transfected into RSF1 KO cells and treated with nocodazole for 16 h. Mitotic cell lysates were subjected to immunoblot analysis with indicated antibodies. **d** Exogenously transfected with Myc-Plk1 WT or CA into RSF1-depleted HeLa cells and mitotic cells were obtained with nocodazole treatment for 16 h. The lysates were processed for immunoblotting analysis using indicated antibodies. **e** Myc-PLK1 WT, KD, or HA-Aurora B expression vectors were introduced into HeLa cells, stably expressing RSF1 shRNA, and then mitotic cells were obtained with nocodazole treatment. The mitotic cells were stained with anti-pAurora B T232 (green) and ACA (red) antibodies, and DAPI was used to stain the nuclei (blue). The graph represents mean ± SEM from three independent experiments; shCtrl *n* = 28, shRSF1 *n* = 34, shRSF1 + PLK1 WT *n* = 37, shRSF1 + PLK1 KD *n* = 29, and shRSF1 + HA-Aurora B *n* = 24 cells. **p < 0.005 vs. control shRNA, ***p < 0.001 vs. *p*cDNA in shRSF1 by two-sided unpaired Student’s *t*-test. Scale bar, 5 μm. Data of **a**, **c**, **d** are representative of three independent experiments. Data of **b** are representative of two independent experiments. Source data are provided as a Source Data file.

 It is reported that PLK1 phosphorylates INCENP and Survivin, and this PLK1-mediated phosphorylation on the CPC component is important for Aurora B docking in the CPC and its activation at the centromere^21,22^. We previously reported that RSF1 at mitotic centromeres provides a compelling docking site for PLK1^33^. Accordingly, not only phospho-Aurora B levels but also the chromatin-bound PLK1 levels were significantly decreased in RSF1-depleted cells (Fig. 2a). Co-immunoprecipitation experiments showed that PLK1 tightly associated with Aurora B and RSF1 in mitotic cells but lost most of the binding ability to Aurora B in the absence of RSF1 (Fig. 2b and Supplementary Fig. 1d). These results suggest that RSF1-mediated PLK1 accumulation at centromeres is important for Aurora B kinase activity. To delineate precisely the major factor regulating the Aurora B activation, we ectopically overexpressed HA-Aurora B, GFP-INCENP, or Myc-PLK1 expression vectors in RSF1 knockout (KO) cells. We hypothesized that ectopic overexpression of the major regulator in RSF1 KO cells would restore Aurora B kinase activity. As shown in Fig. 2c, neither HA-Aurora B nor GFP-INCENP recovered the phospho-Aurora B level in RSF1 KO cells. On the other hand, the phospho-Aurora B levels were fully recovered by exogenous expression of wild-type PLK1 (Myc-PLK1 WT) or a constitutive active form of PLK1 (Myc-PLK1 CA) (Fig. 2d). Consequently, CENP-A phosphorylation by Aurora B kinase was elevated in these cells. The effects of PLK1 were further verified by immunofluorescence staining, which showed PLK1 WT, but not the kinase-dead PLK1 (PLK1 KD) mutant, fully restored the centromeric phospho-Aurora B kinase in RSF1-depleted cells (Fig. 2e). Notably, overexpression of HA-Aurora B by itself did not restore reduced pThr232 level in RSF1-deficient cells. To verify the effect of RSF1-PLK1 axis on Aurora B regulation, we employed two RSF1 mutants (RSF1 S1359A and S1375A), which lost the binding ability to PLK1^33^. Co-immunoprecipitation assay demonstrated RSF1 S1359A or S1375A mutant lost its binding to PLK1 and showed a significant reduction in Aurora B binding (Supplementary Fig. 2a). In addition, reconstitution of phospho-mimetic RSF1 S1375D in RSF1 KO cells enhanced the phosphorylated Aurora B levels, whereas RSF1 S1375A alleviated them. Accordingly, phosphorylation levels on CENP-A remained elevated in cells expressing RSF1 S1375D (Supplementary Fig. 2b). Collectively, these findings support the conclusion that deficient Aurora B kinase activity in RSF1-depleted cells is derived from insufficient PLK1 kinase activity at the centromeres.

### PLK1 induces a novel activating phosphorylation on the Aurora B kinase at Thr236 residue

**Fig. 3:**
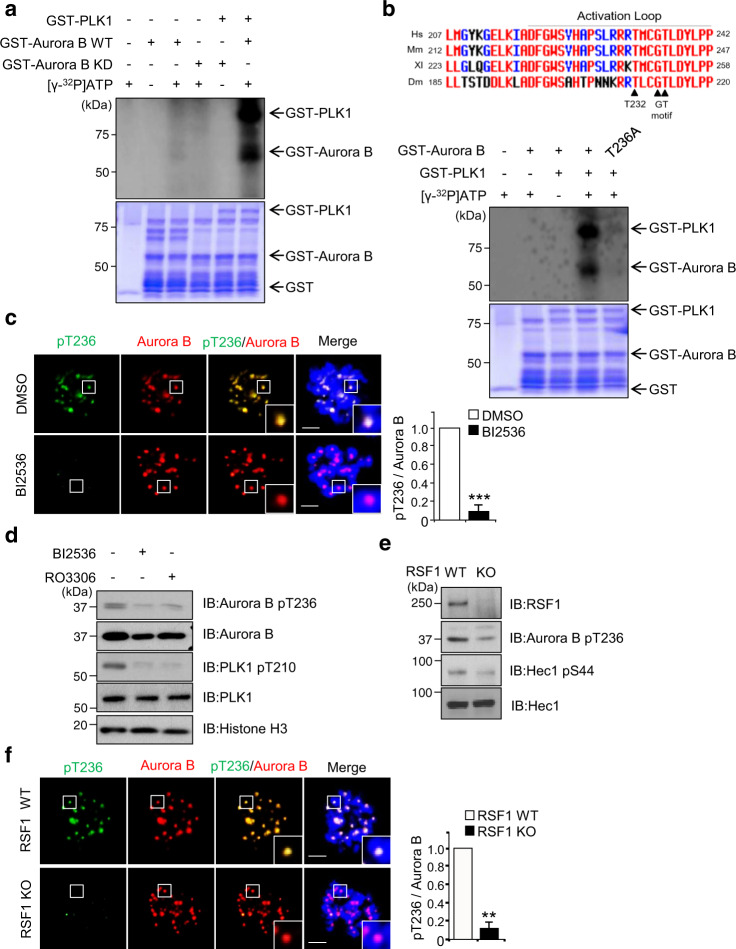
Polo-like kinase 1 modulates Aurora B kinase activity via phosphorylation of GT motif. **a** In vitro kinase assay: recombinant GST-Aurora B WT or KD proteins purified in the bacteria was incubated with the active GST-PLK1 at 30 °C for 30 min in the presence of γ^32^P-ATP. Incorporation of γ^32^P into Aurora B protein was visualized by autoradiography. Coomassie blue staining demonstrates equal protein loading. **b** Alignment of vertebrate Aurora B sequence from humans to mouse across a short region with the conserved GT motif (upper panel). In vitro kinase assay of phosphorylation mutants on the GST-Aurora B were incubated with the active GST-PLK1 at 30 °C for 30 min in the presence of γ^32^P-ATP. **c** Synchronized mitotic HeLa cells were treated with the PLK1 inhibitor BI2536 or DMSO for 30 min. Cells were then fixed and stained with anti-pThr236-Aurora B antibody (green), Aurora B (red), and DAPI (blue). Bars represent mean ± SEM from three independent experiments; DMSO *n* = 32, BI2536 *n* = 35 cells. ***p < 0.001 vs. DMSO by two-sided unpaired Student’s *t*-test. Scale bar, 5 μm. **d** HeLa cells synchronized to mitosis by Nocodazole were pre-treated with MG132 for 1 h and with BI2536, Ro3306, or DMSO for 30 min, followed by western blot analysis with the indicated antibodies. **e** RSF WT or RSF1 KO HeLa cells were synchronized to mitosis by Nocodazole, followed by western blotting analyzed by the indicated antibodies. **f** RSF WT or RSF1 KO HeLa cells were treated with nocodazole for 4 h. Mitotic cells were fixed with 4% paraformaldehyde and stained with anti-pT236-Aurora B (green) and anti-Aurora B (red) antibodies. Nucleus was stained with DAPI (blue). Bar represents mean ± SEM from three independent experiments; WT *n* = 27, RSF1 KO *n* = 44 cells. **p < 0.005 vs. RSF1 WT by two-sided unpaired Student’s *t*-test. Scale bar, 5 μm. Data of **a**, **b**, **d**, **e** are representative of three independent experiments. Source data are provided as a Source Data file.

As PLK1 is crucial in Aurora B kinase activity in early mitosis (Fig. 2), we hypothesized that PLK1 might directly regulate the Aurora B kinase activity in addition to its indirect role of phosphorylating the CPC components^21,22^. In in vitro kinase assay, recombinant GST-PLK1 proteins purified using the baculovirus system were incubated with WT or KD (K106A) GST-Aurora B recombinant proteins and incorporation of ^32^P-ATP was visualized using autoradiography. KD Aurora B proteins did not stimulate incorporation of ^32^P-ATP into Aurora B proteins (Fig. 3a, lane 4) at all. On the other hand, WT Aurora B slightly increased ^32^P-ATP incorporation into Aurora B proteins (Fig. 3a, lane 3), suggesting that Aurora B by itself in vitro contains a very weak autophosphorylation activity. This is consistent with others in that activation of Aurora B through autophosphorylation on Thr232 in vitro requires INCENP or other CPC components^37^. Notably, co-incubation of recombinant PLK1 protein with Aurora B WT protein dramatically increased the incorporation of ^32^P-ATP into Aurora B (Fig. 3a, lane 6). At the same time, a significant increase of ^32^P-ATP incorporation into PLK1 was seen in this assay. We interpreted that the autophosphorylation activity of PLK1 may be enhanced in the presence of Aurora B kinase^38^. In addition, studies on sequence alignment and structural analysis of several mitotic kinase activation segments have delineated well-conserved potential threonine residues for activating phosphorylation within the “GT” motif of the activation segment of mitotic kinases^6^. Multiple sequence alignments showed the autophosphorylation site on Thr232, as well as the phosphorylation site on Thr236, in the GT motif in human Aurora B are well-conserved in higher eukaryotes (Fig. 3b, upper panel). In addition, Thr236 was suggested to be a putative phosphorylation site by PLK1 in other proteomic analysis^39^. Accordingly, the same in vitro kinase assay was carried out using recombinant Aurora B T236A protein. We found that ^32^P-ATP incorporation into recombinant Aurora B T236A proteins was disrupted (Fig. 3b), suggesting that Thr236 in Aurora B is the site that is phosphorylated by PLK1. Meanwhile, we observed that autophosphorylation activity of PLK1 was also suppressed in the presence of Aurora B T236A protein. As PLK1 and Aurora B mutually affect its kinase activity, we speculated that autophosphorylation activity of PLK1 is suppressed in the presence of massively inactive Aurora B protein. Immunofluorescence staining revealed that the pThr236 of Aurora B co-stained with endogenous Aurora B at centromeres of prometaphase cells but it disappeared in the presence of BI2536, a selective inhibitor of PLK1 (Fig. 3c). Inhibition of PLK1 activity by either BI2536 or RO3306, a CDK1 inhibitor, also caused a significant reduction in the pThr236 level of Aurora B in immunoblottings (Fig. 3d). In addition, the same phenomenon was observed in RSF1 KO cells (Fig. 3e, f), which showed a significant loss of the Aurora B-pT236 levels. Together, the data elucidate that the RSF1-PLK1 axis induces a novel phosphorylation on Thr236 of Aurora B.

### Activating phosphorylation on Thr236 of Aurora B is indispensable for the autophosphorylation of Thr232

**Fig. 4:**
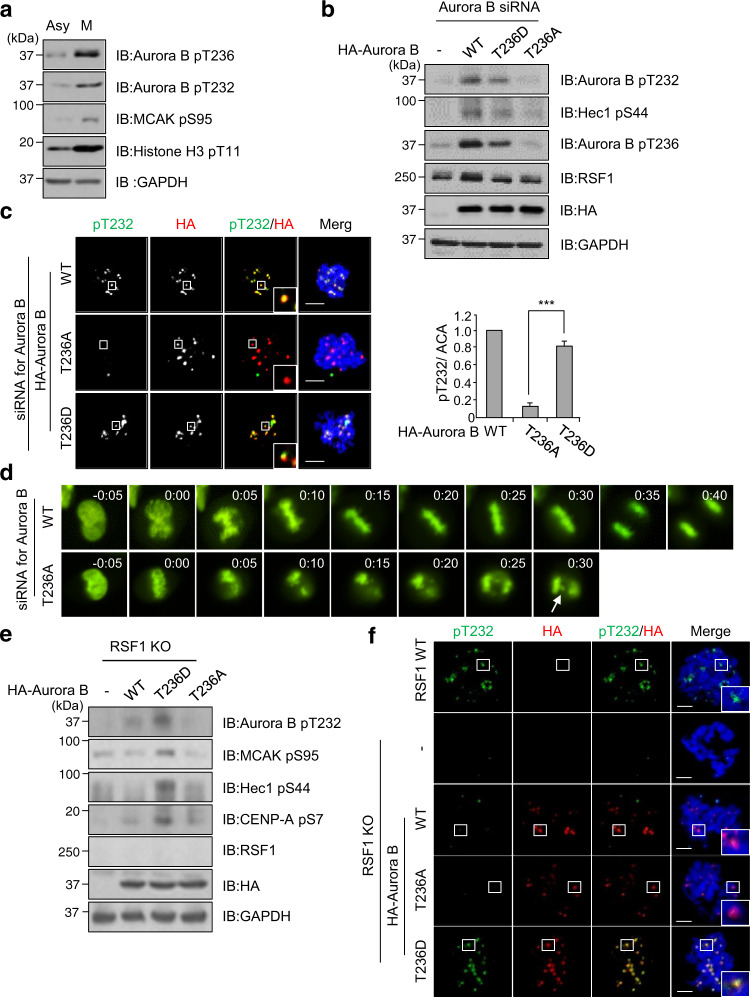
Perturbation of Aurora B phosphorylation on Thr236 causes defects in chromosome segregation. **a** Asynchronously growing HeLa and nocodazole-treated HeLa cells were analyzed by immunoblotting with the indicated antibodies. **b** Immunoblotting of mitotic cell lysates in Aurora B depletion cells after reintroduction of HA-Aurora B WT or T236A/D mutants. **c** Aurora B-depleted HeLa cells were co-transfected with HA-Aurora B WT or T236A/D mutant. Floating mitotic cells were obtained after nocodazole treatment for 4 h and subjected to immunofluorescence images. Images were obtained from representative mitotic cells: pT232-Aurora B (green), HA (red), and DAPI (blue). Statistical analysis of pThr232-Aurora B immunofluorescence intensity at the centromeres. Bars represent mean ± SEM from three independent experiments; WT *n* = 32, T236A *n* = 46, T236D *n* = 39 cells. ***p < 0.001 by two-sided unpaired Student’s *t*-test. Scale bar, 5 μm. **d** H2B-GFP and Aurora B constructs (WT, T236A) were co-transfected in endogenous Aurora B-depleted cells and synchronized at the G1/S boundary by the double thymidine block (DTB) method. Cells were released from DTB for 8 h and carried out time-lapse imaging analysis for 48 h after DTB release. Images were acquired every 5 min. WT *n* = 107, T236A *n* = 180 cells. Scale bar, 5 μm. **e** HA-Aurora B WT or T236A/D mutants were transfected in RSF1 KO cells and analyzed by immunoblotting. **f** Floating mitotic cells were obtained after nocodazole treatment for 4 h and subjected to immunofluorescence image: pT232-Aurora B (green), HA (red), and DAPI (blue). Data of **a**, **b**, **d** are representative of three independent experiments. Data of **e** are representative of two independent experiments. Source data are provided as a Source Data file.

It is widely accepted that phosphorylation of Aurora B at Thr232 in the activating loop is responsible for its kinase activity^35^. We next address how the pThr236 of Aurora B links to its phosphorylation at Thr232. Immunoblot analysis of HeLa cell lysates showed that phosphorylation on the Thr236 and Thr232 residues was detected in nocodazole-arrested mitotic cells but not in asynchronous cells (Fig. 4a). Next, we generated a phospho-dead mutant of Aurora B in which the Thr236 residue was switched to Ala (T236A), as well as a phospho-mimetic mutant T236D (Thr to Asp). We transfected small interfering RNA (siRNA) targeting the 3′-untranslated regions of the *Aurora B* gene into HeLa cells and then overexpressed HA-Aurora B WT or its mutants. Importantly, immunoblot analysis revealed that neither pThr236 nor pThr232 were detected in mitotic cells transfected with Aurora B   T236A mutant, whereas both Aurora B WT and T236D induced a significant phosphorylation on these residues of Aurora B and Hec1 (Fig. 4b). Likewise, the cells expressing either Aurora B WT or Aurora B T236D were immunostained with anti-pT232 antibody. In contrast, the cell expressing the Aurora B T236A mutant was neither immunostained with the anti-pT232 antibody (Fig. 4c) nor with the anti-pMCAK antibody (Supplementary Fig. 3a). These data strongly indicate that phosphorylation on the Aurora B Thr236 by PLK1 is necessary for its subsequent phosphorylation on Thr232. Given the phosphorylation of Aurora B at Thr236 is essential for Aurora B kinase activity, we speculated that the Aurora B T236A mutant would display aberrant mitotic phenotype such as lagging chromosomes. We followed the mitotic progression of HeLa cells after re-expression of HA-Aurora B WT or HA-Aurora B T236A along with a GFP-H2B construct in Aurora B-depleted HeLa cells. Under time-lapse microscopy, 90% of Aurora B WT re-expressing cells exhibited proper chromosome segregation. In contrast, cells transfected with HA-Aurora B T236A often displayed defects in chromosome alignment and segregation leading to lagging chromosomes, which is reminiscent of Aurora B-defective cells (Fig. 4d, arrows). Majority of these cells underwent premature anaphase transition, suggesting that the SAC is compromised (Fig. 4d). However, we also noticed prolonged metaphase in a portion of cells (data not shown). These results correlated with the frequency of lagging chromosome in immunofluorescence staining. Up to 43% of cells expressing HA-Aurora B T236A experienced lagging chromosomes, whereas WT and T236D mutant of Aurora B showed much lower levels of aberrant chromosomes (Supplementary Fig. 3b). Next, we addressed whether a constitutively active form of Aurora B T236D is effective in RSF1 KO cells. Ectopic expression of Aurora B T236D in RSF1 KO cells restored the pT232 level and increased phosphorylation of MCAK, Hec1, and CENP-A (Fig. 4e, f and Supplementary Fig. 3c). However, WT Aurora B in RSF1 KO cells neither restored the pT232 level nor the downstream phosphorylation, because PLK1 activity is still defective in RSF1 KO cells (Fig. 2c). Together, the data demonstrate that phosphorylation on Thr236 of Aurora B is indispensable for the Aurora B kinase activity.

### Activating phosphorylation on Thr236 of Aurora B primes subsequent autophosphorylation of Thr232 on the activation loop

**Fig. 5:**
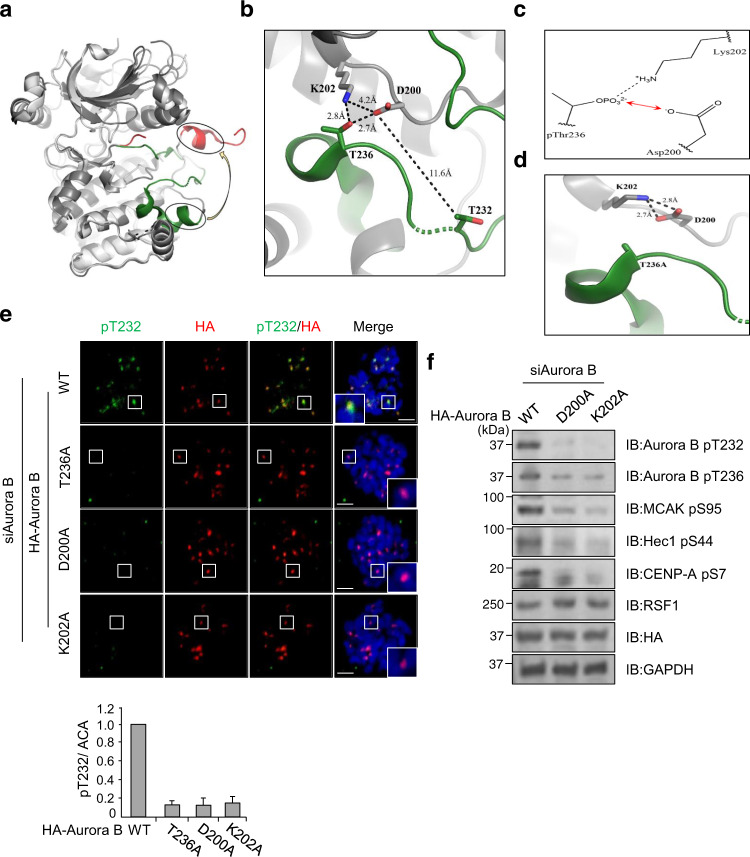
Structural basis of Aurora B autophosphorylation mechanism via the phosphorylation of T236. **a** The superposed crystal structures of human and *Xenopus* Aurora B proteins that are colored in white and gray, respectively. The activation loops in human and *Xenopus* Aurora B are in red and green, respectively. A yellow arrow indicates the positional difference between identical helices highlighted by circles. **b** Structural arrangement of Asp200, Lys202, Thr232, and Thr236 in the crystal structure of the *Xenopus* Aurora B-INCENP complex (PDB code: 2BFX). The activation loop is colored in green. Black dashed lines between residues in sticks represent distances. Nitrogen and oxygen atoms in sticks are colored in blue and red, respectively. **c** The putative interaction mode among Asp200, Lys202, and pThr236. A red arrow represents the electrostatic repulsion, whereas a black dashed line does an electrostatic interaction. **d** The putative interaction mode among Asp200, Lys202, and Ala236 when Thr236 is mutated to alanine. Black dashed lines between residues in sticks represent distances. **e** Floating mitotic cells were obtained after nocodazole treatment for 4 h and subjected to immunofluorescence image: pT232-Aurora B (green), HA (red), and DAPI (blue). Bars represent mean ± SEM from three independent experiments; WT *n* = 22, T236A *n* = 28, D200A *n* = 38, nK202A = 34 cells. Scale bar, 5 μm. **f** HA-Aurora B WT or D200A/K202A mutants were transfected in Aurora B-depleted cells and analyzed by immunoblotting. Data of **f** are representative of two independent experiments. Source data are provided as a Source Data file.

We next used structural modeling to provide a plausible answer on how the phosphorylation on Thr236 affects the autocatalytic activity of Aurora B kinase on the activation loop. The autophosphorylation of Thr232 in Aurora B is the general base-catalyzed reaction (Supplementary Fig. 3) in which the side chain of Asp200 serves as a catalytic base that mediates a nucleophilic attack, which facilitates the transfer of the γ-phosphate to Thr236^40^. The deprotonated hydroxyl group (-O^−^) of Thr232 efficiently performs nucleophilic attack on the γ-phosphate of ATP, generating pThr232. As the activation loop harboring Thr232 and Thr236 is totally disordered in the crystal structure of human Aurora B (PDB code: 4AF3)^37^, we exploited the crystal structure of *Xenopus* Aurora B (PDB code: 2BFX)^41^ to speculate how the phosphorylation of Thr236 facilitates the phosphorylation of Thr232 (Fig. 5a). Asp200 is in the vicinity of Lys202 and Thr236 where Lys202 forms a favorable electrostatic interaction with the pThr236 (Fig. 5b). In contrast, the side-chain carboxylate of Asp200 will conflict with the negative charge of the pThr236 (Fig. 5c). This electrostatic repulsion should perturb the p*K*_a_ value of the side-chain carboxylate of Asp200 for easy protonation that gives an advantage to relieve the electrostatic repulsion. Consequently, Aurora B with the pThr236 facilitates the transfer of proton from Thr232 to Asp200, thus enhancing general base catalysis and subsequent autophosphorylation on Thr232. The autophosphorylation at Thr232 of Aurora B appears to be an intramolecular event, because the Aurora B pThr232 level remained low in immunoprecipitants of the hemagglutinin (HA)-tagged Aurora B mutants (D200A and T236A) even in the presence of WT Aurora B tagged with GST (Glutathione-S-Transferase) (Supplementary Fig. 4c). Thr232 is 11.6 Å away from Asp200 without direct interactions (Fig. 5b). For the intramolecular phosphorylation, therefore, the activation loop containing Thr232 should undergo a substantial conformational change after the phosphorylation of Thr236 to place Thr232 at an adequate position to be activated by Asp200 for the attack of the ATP γ-phosphate. The probability of such a structural alteration induced by the phosphorylation of Thr236 is supported by structural comparison between human Aurora B and *Xenopus* Aurora B. As shown in the superposed structures of the two Aurora B proteins (Fig. 5a), local conformational alterations of the activation loop can occur, maintaining the overall structural integrity. Meanwhile, in Aurora B T236A mutant, the negatively charged carboxylate of Asp200 is highly likely to form a stable ion pair with the neighboring positively charged Lys202. A rotation of the side chain of Lys202 brings it into position to interact with the side-chain carboxylate of Asp200 (Fig. 5d). This stable electrostatic interaction is sure to force the side chain of Asp200 to maintain the carboxylate form. Accordingly, Asp200 cannot efficiently activate/deprotonate the substrate hydroxyl group of Thr232 for nucleophilic attack on the γ-phosphate of ATP. Thus, our model suggests that Aurora B with the phosphorylated Thr236 facilitates the phosphorylation of the Thr232. Next, we verified the functional importance of Asp200 and Lys202 residues in mitotic cells. We established both Aurora B D220A and K202A mutants, and found that expression of these mutants in Aurora B KD cells abolished the activation of aurora B as well as the aurora B-mediated substrate phosphorylation (Fig. 5e, f), strongly suggesting the importance of these residues in the activation of Aurora B kinase. Taken together, this is the first data presenting an activating phosphorylation of Thr236 in the Aurora B kinase and structural interpretation of its catalytic effect on subsequent phosphorylation at Thr232.

### PLK1 separately conducts two key events of phosphorylation on Aurora B or BubR1 depending on MT–KT attachment

**Fig. 6:**
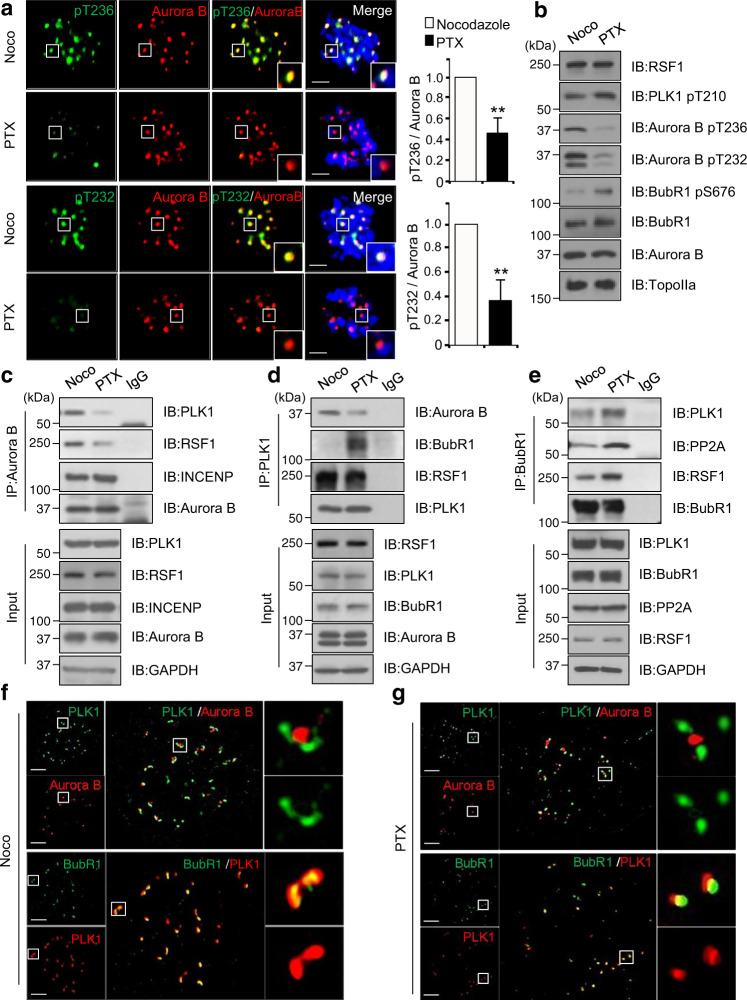
Precise regulation of RSF1-PLK1 axis ensure proper kinetochore bi-orientation. **a** HeLa cells were treated with nocodazole or paclitaxel treated for 4 h. Mitotic cells were fixed with 4% paraformaldehyde and stained with anti-pT236 or anti-pT232-Aurora B (green) and anti-Aurora B (red) antibodies. Nucleus was stained with DAPI (blue). The bar graph represents mean ± SEM from three independent experiments; signal intensity of Aurora B-pT232 (Noco *n* = 26, PTX *n* = 31) or pT236 (Noco *n* = 23, PTX *n* = 36) at the kinetochores. **p < 0.005 vs. Nocodazole by two-sided unpaired Student’s *t*-test. Scale bar, 5 μm. **b** Immunoblotting of chromatin fractions in nocodazole- or paclitaxel-treated HeLa cells using indicated antibodies. **c** Nocodazole- or paclitaxel-treated HeLa cells were immunoprecipitated using anti-Aurora B antibodies. The inputs and immunoprecipitants were analyzed by western blotting. **d** Mitotic extracts from nocodazole- or paclitaxel-treated HeLa cells were immunoprecipitated using anti-PLK1 antibody. **e** Nocodazole- or paclitaxel-treated HeLa cells were immunoprecipitated using anti-BubR1 antibody and immunoprecipitated samples were immunoblotted with PP2A antibodies. **f** Super-resolution confocal single slice images of PLK1 in prometaphase cells. Nocodazole-treated HeLa cells were fixed with 4% paraformaldehyde and stained with indicated antibodies. Scale bars, 5 μm. **g** Super-resolution confocal images of PLK1 in paclitaxel-treated prometaphase cells. Floating mitotic cells were stained with indicated antibodies. Scale bars, 5 μm. Data of **b**–**e** are representative of three independent experiments. Source data are provided as a Source Data file.

So far, we demonstrated that the RSF1-PLK1 axis acts as a strong activator of the Aurora B kinase. In higher eukaryotes, PLK1 and Aurora B show potentially antagonistic activities on stability of mitotic spindles in early mitosis^23^. To correct erroneous MT–KT attachment, Aurora B kinase phosphorylates the Ndc80 complex proteins, decreasing the MT-binding affinity to the KT^42^. In contrast, PLK1 increases the stability of MT–KT interactions through antagonizing this Aurora B-mediated phosphorylation by recruitment of PP2A-B56 phosphatase. PLK1 phosphorylates the Ser676 residue of BubR1 at KTs, which serves as a docking site for PP2A-B56^10,18^. Thus, the key question is how RSF1 reconciles the antagonistic activities between PLK1 and Aurora B within the same regulatory axis. We hypothesized that the RSF1-PLK1 axis may work two key events of PLK1-mediated Aurora B phosphorylation (Figs. 1 and 2) and BubR1 phosphorylation^33^ separately in a spatiotemporal manner within centromeres and KTs. To test it, we first used the kinesin-5 inhibitor monastrol to create monopolar spindles and syntelic attachments that can be corrected by active Aurora B. Then the proteasome inhibitor MG132 was briefly treated to the cells released from monastrol treatment, allowing progression to metaphase with bi-oriented KT–MT attachments. The pThr236 (Supplementary Fig. 5a) and pThr232 (Supplementary Fig. 5b) of Aurora B, as well as the phospho-MCAK (Supplementary Fig. 5c), strongly showed up at monopolar spindles. After release from monastrol, the phosphorylated Aurora B and MCAK disappeared from aligned chromosomes (Supplementary Fig. 5, inset 2), whereas they stayed on the unaligned chromosomes (Supplementary Fig. 5, inset 1). As aligned chromosomes at metaphase plate are balanced by bi-oriented MT–KT attachment, we next analyzed cells arrested at prometaphase by treatment with MT-destabilizing nocodazole or MT-stabilizing paclitaxel (Fig. 6a). As expected, there was a considerable accumulation of pThr236 and pThr232 of Aurora B in nocodazole-arrested cells. In contrast, the phosphorylation was significantly lower in paclitaxel-arrested cells. As tension was eliminated in paclitaxel-arrested cells even with full MT–KT attachment, differences in phosphorylation on Aurora B would come from the extent of MT–KT attachment, not from the tension. The same results were found in immunoblot analysis in HeLa cells arrested at prometaphase by treatment with nocodazole or paclitaxel (Fig. 6b and Supplementary Fig. 6a). In both treatments, PLK1 pT210 levels remained phosphorylated, but exhibiting higher levels in paclitaxel-treated cell. The pThr236 and pThr232 were shown up only in nocodazole-treated cells. In contrast, the PLK1-induced phosphorylation on Ser676 of BubR1 was not observed in nocodazole-treated cells, whereas it was significantly elevated in paclitaxel-arrested cells (Fig. 6b and Supplementary Fig. 6b). Thus, the data suggested that PLK1 preferentially selects the phosphorylation target of Aurora B or BubR1 based on the MT attachment. In fact, PLK1 binding to Aurora B and BubR1 were changed in these two different conditions. Aurora B binding to PLK1 remained stable in nocodazole-treated cells but a significant reduction was found in paclitaxel-treated cells (Fig. 6c). In both treatments, Aurora B interaction with the CPC component of INCENP was not altered. A reciprocal immunoprecipitation with anti-PLK1 antibody also elucidated a reduction in PLK1 binding to Aurora B in paclitaxel-arrested cells (Fig. 6d). On the contrary, PLK1 binding to BubR1 was not observed in nocodazole-arrested cells but showed a significant increase in paclitaxel-arrested cells. When immunoprecipitation with anti-BubR1 antibody was carried out, BubR1 binding to PLK1 and to PP2A were higher in paclitaxel-treated cells than in nocodazole-treated cells (Fig. 6e). A spatial regulation of PLK1 on Aurora B and BubR1 was also verified by analyzing high-resolution confocal imaging of PLK1 and Aurora B localization under super-resolution microscopy (SIM). Aurora B in the CPC stably locates at inner centromeres and insoluble BubR1 is found at outer KTs^11,43,44^. In nocodazole-arrested cells, PLK1 formed a comet (dumbbell)-like staining that partially overlapped with Aurora B located at the inner centromeres (Fig. 6f). Consistent with the finding above (Fig. 6c, d), PLK1 was largely separated from Aurora B in paclitaxel-arrested cells (Fig. 6g), showing redistribution of PLK1 from the centromeres to KTs. Double-immunostaining of BubR1 and PLK1 displayed the PLK1 staining overlapped with BubR1 in nocodazole-arrested cells. In paclitaxel-arrested cells, PLK1 no longer showed a comet-like signal but fully overlapped with BubR1 signal (Fig. 6g). All these data strongly support our hypothesis that PLK1 separately conducts two key events of phosphorylation on Aurora B or BubR1 depending on MT–KT attachment.

### RSF1-PLK1 movement to KTs is regulated by the Aurora B-mediated phosphorylation on centromeric histone H3

**Fig. 7:**
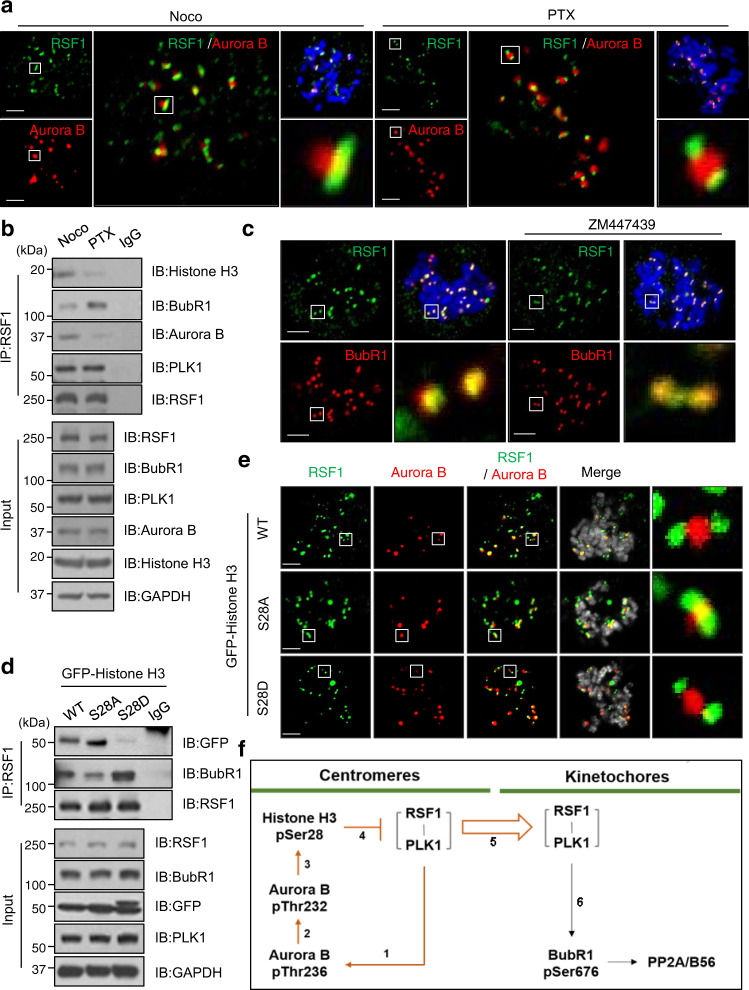
RSF1 localization to mitotic kinetochores requires the phosphorylation of histone H3 on Ser28. **a** HeLa cells were treated with nocodazole or paclitaxel for 4 h and prometaphase cells were stained with anti-RSF1 (green) and anti-Aurora B (red) antibodies. Scale bars, 5 μm. **b** Nocodazole- or paclitaxel-treated HeLa cells were immunoprecipitated using anti-RSF1 antibodies. The inputs and immunoprecipitants were analyzed by western blotting. **c** HeLa cells were treated with paclitaxel treated for 4 h and prometaphase cells were pre-treated with Aurora B inhibitor ZM447439 for 30 min in the presence of MG132. Mitotic HeLa cells were stained with anti-RSF1 (green) and anti-BubR1 (red) antibodies. Scale bars, 5 μm. **d** HeLa cells were transfected with indicated GFP-Histone H3 WT or S28A/D constructs and treated with paclitaxel for 16 h. Mitotic cell lysates were followed by immunoprecipitation using anti-RSF1 antibody. **e** HeLa cells were introduced with GFP-Histone H3 WT or S28A/D mutants and treated with paclitaxel for 4 h. Images were obtained from representative mitotic cells: RSF1 (green), Aurora B (red), and GFP (gray). Scale bars, 5 μm. **f** A proposed model for spatiotemporal regulation of the on/off switch on Aurora B kinase by the RSF1-PLK1 axis. New findings are colored in orange lines. Data of **b**, **d** are representative of three independent experiments. Source data are provided as a Source Data file.

So, the next question is how the RSF-PLK1 axis conducts the selective phosphorylation of Aurora B or BubR1 within centromeric regions. As RSF1 recruits PLK1 to the centromeric region^33^, we examined whether RSF1 showed the positional change such as PLK1 depending on MT attachment. Under confocal microscope, co-immunostaining of RSF1 and Aurora B showed differences in their relative positions depending on MT attachment (Fig. 7a). In nocodazole-arrested cells, RSF1 aligned right next to Aurora B, which is similar to co-staining of PLK1 and Aurora B, although PLK1 is more apart from Aurora B than RSF1. On the other hand, two RSF1 spots were separated by the centromeric Aurora B in paclitaxel-arrested cells, which is alike to PLK1 (Fig. 7a). The same conclusion was obtained in co-immunoprecipitation experiments (Fig. 7b). RSF1 associated with histone H3 and Aurora B in nocodazole-treated cells, whereas it bound BubR1 better at KTs in paclitaxel-arrested cells (Fig. 7b). Thus, the results indicate that both RSF1 and PLK1 are redistributed from the centromeres to KTs depending on MT attachment. Next, we asked whether any posttranslational modifications of histones at centromeric nucleosomes might alter interaction of RSF1 with centromeric nucleosomes. To test it, we screened interactions of RSF1 with histones using histone peptide microarray, which contains the posttranslationally modified histone peptides at different amino acid resides. Interestingly, RSF1 remained its binding to most of histone peptides, except the phosphorylated histone H3 on Ser28 (Supplementary Fig. 7a, b), which is identified as a phosphorylation target of Aurora B^45^. Indeed, immunofluorescence staining verified the histone H3 pSer28 at centromeres that disappeared in the presence of ZM447439. (Supplementary Fig. 7c). The result also raises a possibility that the Aurora B-mediated phosphorylation on Ser28 of histone H3 is the que for re-localization of RSF1 to KTs. Indeed, two RSF1 spots were overlaid with BubR1 in paclitaxel-arrested cells, whereas treatment with ZM447439 suppressed the redistribution of RSF1 to KTs (Fig. 7c), showing the remaining RSF1 at the centromeres. Next, we generated the histone H3 S28A and S28D mutants, and co-immunoprecipitation assay revealed that the phosphorylation mimetic mutant of H3 S28D significantly reduced the RSF1 binding (Fig. 7d). In contrast, RSF1 bound BubR1 better when the H3 S28D was expressed, suggesting re-localization of RSF1 to KTs. Likewise, RSF1 remained on the centromeres in cells expressing histone H3 S28A, whereas it moved to KTs in cells introduced with histone H3 S28D (Fig. 7e) in immunofluorescence staining analysis. Together, the data elucidate that the activated Aurora B by the RSF1-PLK1 axis at centromeres phosphorylates the centromeric histones, which triggers the re-localization of the RSF1-PLK1 to KTs. This feedback loop between centromeres and KTs reconciles antagonistic activities between Aurora B and PLK1 in early mitosis.

## Discussion

The RSF1 chromatin remodeler is tightly associated with CCAN components and its presence at mitotic centromeres is essential for faithful chromosome alignment and segregation^33,34^. We here uncovered a central role of the RSF1-PLK1-Aurora B circuit in early mitosis (Fig. 7f and Supplementary Fig. 8). The RSF1-PLK1 axis created a novel phosphorylation on Aurora B in the activation loop (①). This phosphorylation precedes the autophosphorylation of Aurora B at Thr232 (②). Thus, PLK1 is a key activating kinase on Aurora B that destabilizes erroneous MT attachment. Aurora B induces the phosphorylation on centromeric histone H3 on Ser28 (③), which suppresses the RSF1 binding to centromeric components or histones (④). RSF1-PLK1 now positions at KTs (⑤). As previously characterized^17–19^, PLK1 at KTs phosphorylates BubR1, which contributes to accumulation of PP2A-B56 through its direct interaction (⑥). This transition would be important for PLK1-mediated stabilization of MT–KT attachment. Thus, spatiotemporal separation of Aurora B and PLK1 activities may efficiently coordinate the MT detachment/attachment in early mitosis. Our data here provide key insights into centromeric signaling network in early mitosis.

Activation of Aurora B kinase in the CPC is regulated by multiple regulatory steps. Aurora B activation is initiated by binding to the INCENP, which molds and stabilizes the active conformation of the G-loop of Aurora B, opening its catalytic cleft. Then, Aurora B phosphorylates the Thr-Ser-Ser motif on the IN box of INCENP and this phosphorylation is suggested to allow a fully active Aurora B kinase accompanied by autophosphorylation of Aurora B at Thr232^35,37,42^. At present, the crystal structure of Aurora B is only available for the partially active form, but many protein kinases share a similar activation mechanism such that one or multiple phosphorylation sites in the activation loop are important for activation. MTs and TD-80, an inner centromeric protein, are suggested to lead to activation of the Aurora B kinase pathway, possibly through *trans*-autoactivation and/or yet unknown allosteric mechanisms^43–45^. Aurora B activation is also controlled by extrinsic mechanisms of other mitotic kinases that phosphorylate INCENP, Borealin, and Survivin of the CPC complex^37,42,46,47^. Interaction and phosphorylation of the CPC components by MPS1, HASPIN, and PLK1 appear to promote centromere recruitment of the CPC through histone mark recognition and Aurora B activation^21,48–51^. We here showed the direct phosphorylation on Thr236 residue of Aurora B by PLK1 (Figs. 3 and 4). This phosphorylation occurs at the GT motif on the activation segment of Aurora B. The initial phosphorylation on Thr236 by PLK1 caused Aurora B to be autophosphorylated on the Thr232 residue, which is the classic site on the activation loop. Our structural modeling provides an insight that Asp200 acts as the catalytic base for the autophosphorylation on Thr232 (Fig. 5). In an active kinase when it is poised for transferring a phosphate from ATP to a substrate protein, Thr236 is thought to be part of the active site priming the substrate protein for accepting the phosphate. When Thr236 is phosphorylated, it would be incompatible with the position indicated in the presence of ATP. Most plausibly when Thr236 is phosphorylated it will most likely interact with the adjacent HRD (His-Arg-Asp)  arginine, which precedes the catalytic base, Asp200. The conformation of the activation segment will be ordered differently or will simply be very dynamic as it undergoes autophosphorylation on Thr232. Once Thr232 is phosphorylated, it can interact with both the HRD Arg and with the C Helix where it mediates cross-talk between the N-lobe and the C-lobe. In this case, however, interaction of Thr236 with the HRD motif would be hampered by its phosphorylation. Accordingly, pThr236 might be either dephosphorylated by phosphatase or the pThr236 would significantly change the structure of P + 1 loop, allowing substrate recognition. This is a novel mechanism and is likely to be important for other kinases, because this GT motif is shared by most protein kinases^6^.

How tension and/or MT attachment status is sensed and translated into the regulation of Aurora B kinase activity is a lingering question. Tension-dependent physical separation of Aurora B from its KT substrates has been proposed^11,52,53^. In this model, Aurora B remains at the inner centromeres and therefore, it does not reach its KT substrates under tension. On the other hand, MT-dependent Aurora B regulation is suggested. In this model, the force generated by MT–KT attachment is transduced to the CPC, which regulates Aurora B activity. Thus, tension is a critical factor that regulates Aurora B activity. Our study here showed that in the absence of tension, Aurora B kinase activity is regulated by the MT–KT attachment (Fig. 6). Under conditions of the spindle poisoning drugs nocodazole and paclitaxel, tension does not exist. We found that the phosphorylation status of Thr236 and Thr232 of Aurora B was significantly reduced by paclitaxel treatment where MT depolymerization was suppressed. We interpreted that this was driven by translocation of PLK1 to KTs (Fig. 6f) and, thus, PLK1 no longer phosphorylated the Thr236 of Aurora B (Fig. 6b). Thus, the data here elucidated that the Aurora B kinase activity turns off when full MT–KT attachment is achieved. This is supported by the finding that Aurora B-pThr236 level was dimmed out in the metaphase cell (Supplementary Fig. 6a). In addition, the activated Aurora B at the centromeres can be counteracted by a centromeric pool of PP2A-B56 phosphatase^20^. We here propose that RSF1 as a centromere-interacting protein is a crucial regulatory component in the control of PLK1 and Aurora B kinase activities, independent of its chromatin remodeling activity.

## Methods

### Cell lines and cell culture conditions

The HeLa human cervical cancer cell line was obtained from the American Type Culture Collection (CCL-2). HeLa cells were maintained in high-glucose Dulbecco’s modified Eagle’s medium supplemented with 10% fetal bovine serum (Invitrogen). All cell lines were confirmed free of mycoplasma contamination by MycoFluor^TM^ Mycoplasma Detection Kit (Invitrogen, #M7006). To obtain cells synchronized at prometaphase, cells were treated with 100 ng/ml of paclitaxel (Sigma-Aldrich) or 100 nM of nocodazole (Sigma-Aldrich) for 12–16 h and collected by gentle shake-off.

### Antibodies and reagents

A list of all antibodies used in this study is provided in the Supplementary Table 1. The following reagents were used: Aurora B inhibitor ZM447439 (Tocris), CDK inhibitor RO3306 (Enzo), Nocodazole (sigma), Paclitaxel (Tocris), PLK1 inhibitor IB2536 (Axonmedchem), and Puromycin Dihydrochloride (Invitrogen).

### Generation of TALEN RSF1 KO cell lines

RSF1 KO HeLa cells were generated by transcription activator-like effector nucleases (TALEN)-mediated genome engineering and the RSF1-specific TALEN plasmids composed of the fluorescence surrogate reporter (pRG2S) system were purchased from ToolGen, Inc. (Korea). At 48 h post transfection of RSF1-specific TALEN plasmids with pRG2S, the cells expressing green fluorescence were sorted under fluorescence-activated cell sorting (FACS Vantage, BD Biosciences).

### Plasmid construction, mutagenesis, and transfection

A list of all PCR primers used in this study is provided in the Supplementary Table 2. The plasmid encoding human Aurora B WT or KD were cloned into the pGEX6p-1 vector to produce N-terminal GST fusion proteins. Site-directed mutagenesis was carried out on the GST-Aurora B plasmid using Muta-Direct™ Site Directed Mutagenesis Kit (iNtRON Biotechnology) to change Thr236 and Ser 22, Ser 62, and Thr 64 to Arg or Asp. These vectors were transformed into bacteria and the expression of GST-Aurora B proteins was purified from bacterial lysates using Glutathione Sepharose 4B (GE Healthcare)^52^. HA-tagged full-length constructs of Aurora B were kindly provided by Chang-Woo Lee (Sungkyunkwan University). The transfection of plasmids or siRNA oligonucleotides was carried out using polyethylenimine (Polysciences) or Lipofectamine 2000 (Invitrogen) following the manufacturer’s protocol.

### Immunoprecipitation and in vitro binding assays

For immunoprecipitation, cells were lysed using a sonicator (EpiShear™ Probe Sonicator, Active motif, 120 Watt, 20 kHz) in E1A buffer (50 mM Tris-HCl pH 7.4, 150 mM NaCl, 0.1% NP-40, 1 mM dithiothreitol (DTT), 5 mM EDTA) containing protease and phosphatase inhibitors (1 mg/ml aprotinin, 1 mg/ml leupeptin, 5 mM NaF, 0.5 mM Na_3_VO_4_). Protein lysates were immunoprecipitated with indicated antibodies for 12 h at 4 °C under constant rotation. The protein–antibody complex was further incubated with protein A-Sepharose beads (GE Healthcare) for 1 h 30 min at 4 °C and the immune complex was washed and subjected to immunoblotting. For in vitro binding assays, recombinant proteins of GST-Aurora B WT, GST-Aurora B KD, GST-PLK1, and His-PLK1 were prepared as described above. The proteins were incubated for 2 ~ 12 h at 4 °C under constant rotation and beads-bound immune complexes were washed for four times and subjected to immunoblot analysis.

### In vitro kinase assay

Recombinant GST-fused Aurora B WT or KD proteins were incubated with either human GST-PLK1 in the kinase buffer (20 mM HEPES, 0.14 M NaCl, 3 mM KCl, 5 mM MgCl_2_ pH 7.4) with 2 μCi of γ^32^P‐ATP (PerkinElmer^TM^) for 30 min at 30 °C. The reactions were terminated by adding 6× SDS sample buffer followed by heating 100 °C for 5 min. The proteins were separated on gradient SDS-polyacrylamide gel and the incorporation of ^32^P was visualized by autoradiography.

### Cell fractionation

Mitotic cells were lysed with fractionation buffer I (10 mM Tris pH 8.0, 25 mM KCl, 5 mM MgCl_2_, 0.5% NP-40, 1 mM DTT, 1 mg/ml aprotinin, 1 mg/ml leupeptin, 5 mM NaF, 0.5 mM Na_3_VO_4_) and incubated on ice for 5 min. The supernatant (S1) and pellet (P1) were obtained after centrifugation at 1300 × *g* for 5 min at 4 °C. The S1 fraction was refined by high-speed centrifugation at 20,000 × *g* for 10 min at 4 °C and the supernatant was used as a soluble cytosolic fraction (S2). The insoluble pellet (P1) was washed twice and lysed with fractionation buffer II (10 mM Tris pH 8.0, 500 mM NaCl, 0.1% NP-40, 5 mM EDTA, 1 mg/ml aprotinin, 1 mg/ml leupeptin, 5 mM NaF, 5.5 mM Na_3_VO_4_) by sonication (Active motif, 120 W, 20 kHz). After centrifugation at 1700 × *g* for 5 min at 4 °C, the chromatin-bound nuclear fraction (supernatant) was obtained. The concentrations of lysates were normalized by Bradford assay (Bio-Rad Laboratories) and lysates were analyzed by immunoblotting.

### Immunostaining

HeLa cells were treated with 100 ng/ml of nocodazole for 4 h and floating mitotic cells were fixed with 4% paraformaldehyde for 15 min and then permeabilized with 0.5% Triton X-100 for 10 min. The fixed cells were blocked with 5% bovine serum albumin for 1 h at room temperature (RT). For image acquisition, Nikon A1R-A1 Confocal Microscope system with ×60 1.4 NA Plan-Apochromat objective (Nikon Instrument, Inc.) or LSM710 with ×63× 1.4 NA Plan-Apochromat objective (Carl Zeiss), Nikon AIR HD25 with N-SIM at the Three-Dimensional Immune System Imaging Core Facility of Ajou University. Images were used and analyzed by the NIS elements C program or the ZEN 2011 program, and image processing and quantification were carried out with ImageJ.

### Live-cell imaging

HeLa cells transfected with GFP-H2B and other appropriate plasmids were synchronized at the G1/S boundary by the double thymidine block method as described previously. At 8 h after release from the thymidine block, the cells were treated with 9 mM of RO3306 for 2 h to arrest cells at G2 phase. The synchronized HeLa cells at G2 phase regained the cell cycle progression in a microscope stage incubator at 37 °C in a humidified atmosphere of 5% CO_2_ throughout the experiment. Fluorescence images were acquired every 5 min using a Nikon Eclipse Ti with a ×20 1.4 NA Plan-Apochromat objective. Images were captured with an iXonEM þ897 Electron Multiplying charge-coupled device camera and analyzed using NIS elements Ar microscope imaging software.

### Aurora B structure modeling

Structural analysis and display were performed using PyMOL (https://www.pymol.org/2).

### Histone peptide array

MODified^TM^ Histone Peptide Array is now commercially available from Active Motif (catalog number 13001). The array was blocked by incubation in TTBS buffer (10 mM Tris/HCl pH 7.5, 0.05% Tween-20, and 150 mM NaCl) containing 5% non-fat milk for 1 hr at RT, then washed two times with TTBS buffer, one time with interaction buffer (100 mM KCl, 20 mM HEPES pH 7.5, 1 mM EDTA, 0.1 mM DTT, and 10% glycerol), and incubated with purified GST-tagged RSF1 protein (2 μg/mL) at 4 °C for 8 h in interaction buffer. After washing with TTBS buffer three times, the array was incubated with monoclonal anti-RSF1 (1 : 1000) in TTBS buffer containing 1% non-fat dry milk for 2 h at RT. Then, the membrane was washed three times with TTBS and incubated with secondary anti-rabbit Alex-fluor 647 (1 : 5000) for 1 h at RT, and then washed three times with TTBS buffer. Array image was scanned with GenePix 4000B (Axon Instruments, Union City, CA).

### Statistical analysis

In each result, the error bars represent the mean ± SEM from at least three independent experiments. The statistical significance was determined with two-sided unpaired Student’s *t*-test. *P*-value < 0.05 was considered statistically significant. Significance is indicated by asterisk (*P < 0.01, **P < 0.005, ***P < 0.001 vs. the control).

### Reporting summary

Further information on research design is available in the Nature Research Reporting Summary linked to this article.

## Supplementary information

Supplementary Information

Reporting Summary

## Data Availability

Data are available in the article or Supplementary Information. Source data are provided with this paper.

## Source data

Source Data
