# Supplementary material for: Spatiotemporal coordination of the RSF1-PLK1-Aurora B cascade establishes mitotic signaling platforms
Source: Nat Commun. 2021 Oct 11;12:5931. doi: 10.1038/s41467-021-26220-z (PMC8505570; doi:10.1038/s41467-021-26220-z)
Supplement: Supplementary file 1 — Supplementary Information [file 41467_2021_26220_MOESM1_ESM.pdf]

Supplemental Information

Spatiotemporal Coordination of the RSF1-PLK1-Aurora B Cascade Establishes Mitotic Signaling Platforms

Ho-Soo Lee, Sunwoo Min, Ye-Eun Jung, Sunyoung Chae, June Heo, Jae-Ho Lee, TaeSoo Kim, Ho-Chul Kang, Makoto Nakanish, Sun-Shin Cha, Hyeseong Cho

Supplementary Figure 1

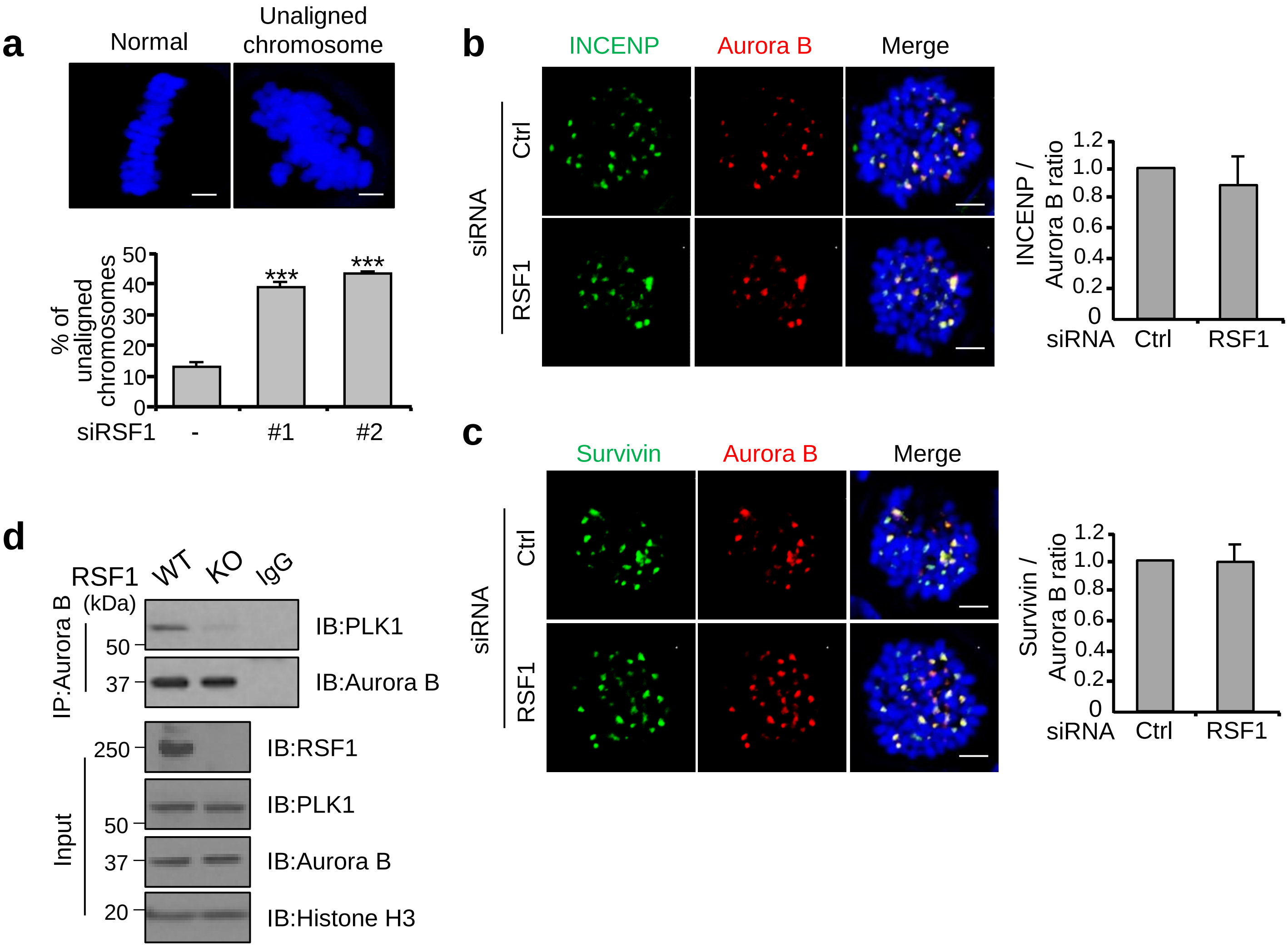

Supplementary Figure 1 The centromeric localization of CPC components were not altered in RSF1 depleted cells **(a)** After transfection with RSF1 siRNA for 48 h, HeLa cells were treated with 100 ng/ml nocodazole for 12 h and released into the media containing 20 μM MG132 for 2 h. These cells arrested at metaphase were fixed with 4% paraformaldehyde and stained with DAPI (blue). Bars represent mean ± SEM from three independent experiments; n_{siCtrl} = 232, $n_{siRSF1\#1}$ = 247, $n_{siRSF1\#2}$ = 229 cells. Statistical significance was tested by two-sided t test; $^{***}p < 0.001$. Scale bar, 5 μm. **(b)** Mitotic cells were fixed with 4% paraformaldehyde and stained with INCENP (green) and Aurora B (red) antibodies. Nucleus was stained with DAPI (blue). The graph represents quantification of INCENP signal intensity (right panel). Bars represent mean ± SEM from three independent experiments; n_{siCtrl} = 24, n_{siRSF1} = 34 cells. Scale bar, 5 μm. **(c)** Mitotic cells were stained with Survivin (green) and Aurora B (red), DAPI (blue). The quantification of signal intensity of Survivin (bottom panel). Bars represent mean ± SEM from three independent experiments; n_{siCtrl} = 21, n_{siRSF1} = 27 cells. Scale bar, 5 μm. **(d)** RSF1 WT or RSF1 KO mitotic cell lysates were immunoprecipitated with anti-Aurora B antibody and immunoblotted with indicated antibodies. Data of d are representative of three independent experiments. Source data are provided as a Source Data file.

Supplementary Figure 2

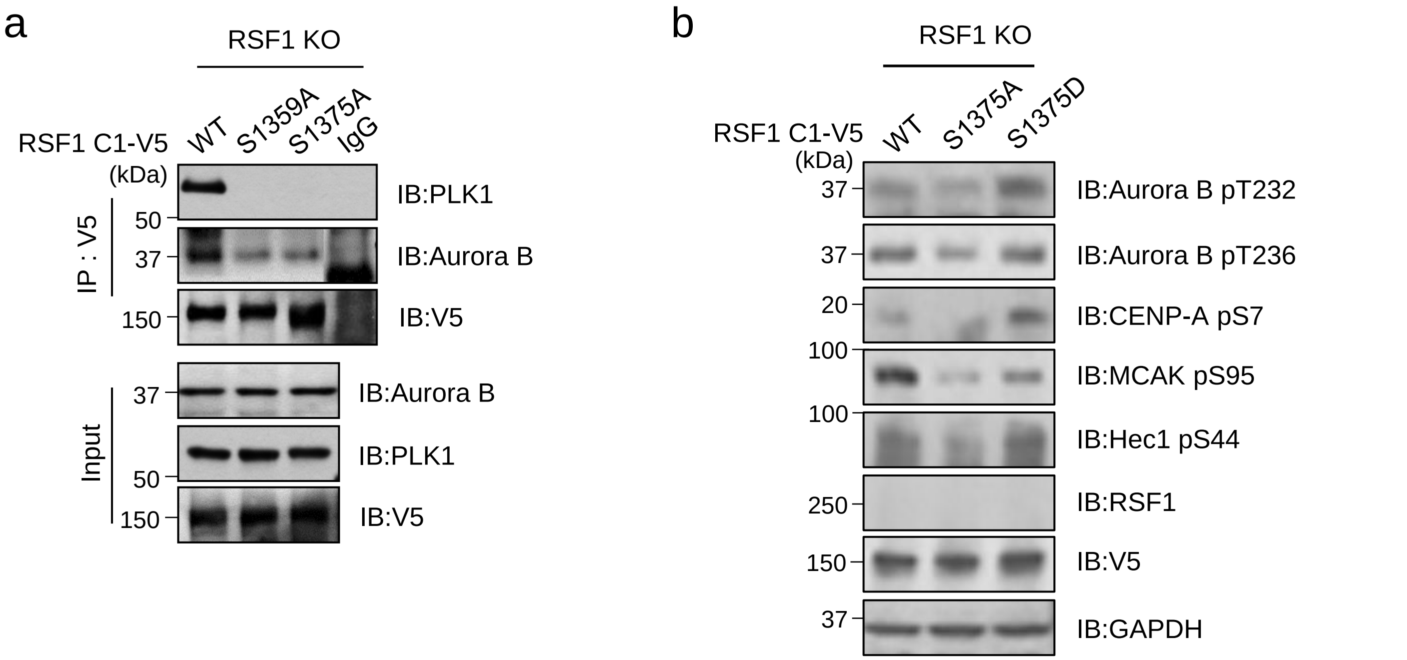

Supplementary Figure 2 Phosphorylation at S1375 of RSF1 is required for PLK1 binding and Aurora B kinase activity. **(a)** RSF1-C1-V5 plasmid were transfected in RSF1 KO cells and co-immunoprecipitation experiments were carried out with an anti-V5 antibody. **(b)** RSF1 KO cells were introduced with indicated RSF1 constructs and treated with nocodazole for 16h. Mitotic cell lysates were analyzed by immunoblotting. Data of a, b are representative of two independent experiments.

Supplementary Figure 3

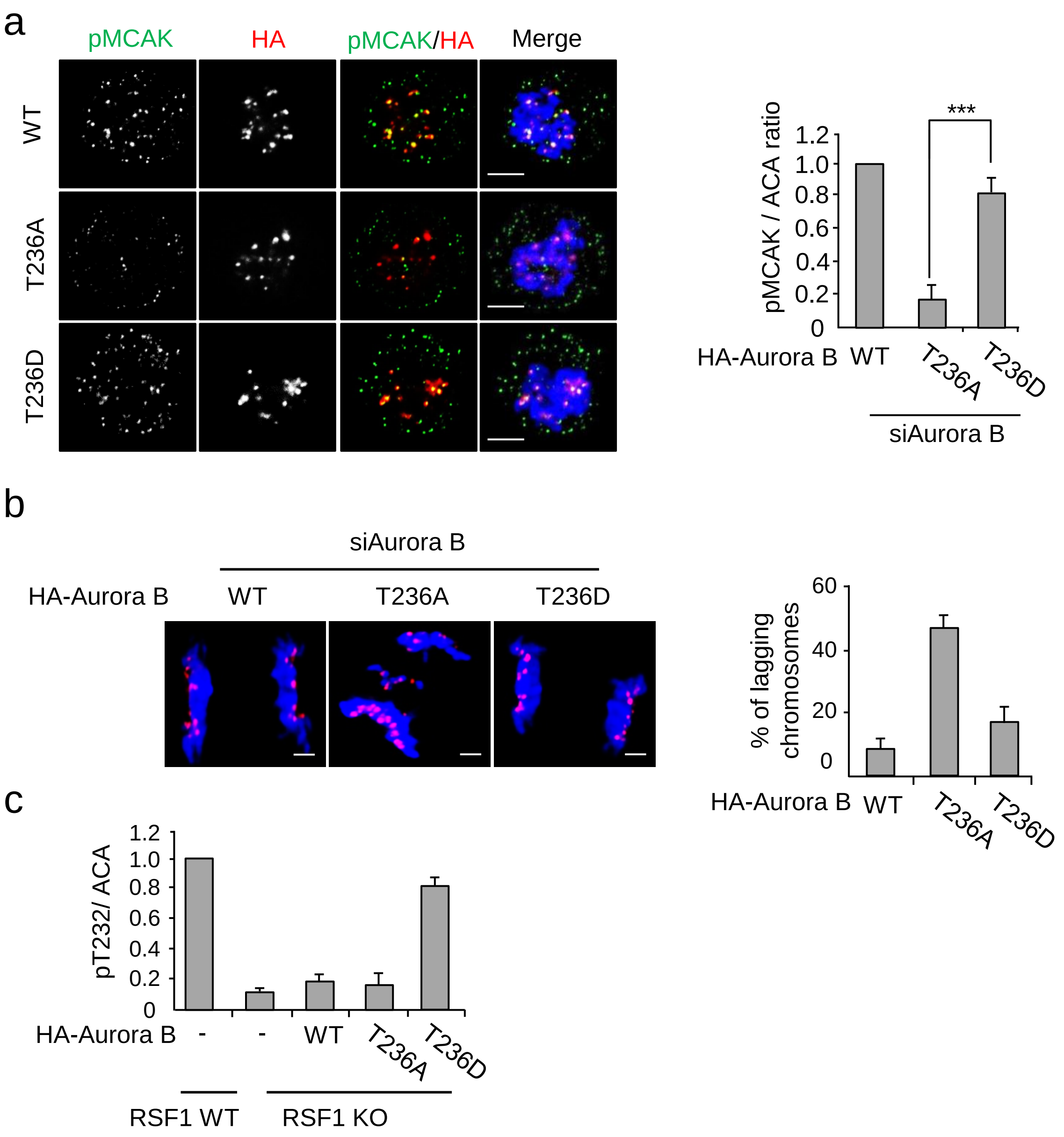

Supplementary Figure 3 Aurora B T236 phosphorylation is required for Aurora B kinase activity **(a)** Images of HeLa cells transfected with Aurora B T236A or T236D mutants. Mitotic HeLa cell were fixed and stained with anti-MCAK pS95 antibody (green), anti-HA antibody (red), and DAPI (blue). Bar graphs represent the fluorescence ratio of pMCAK against ACA staining (right panel). Data represent mean ± SEM from three independent experiments; $n_{WT} = 23$, $n_{T236A} = 27$, $n_{T236D} = 31$ cells. Statistical significance was tested by two-sided t test; ***p < 0.001. Scale bar, 5 μm. **(b)** HeLa cells were co-transfected with HA-Aurora B WT or T236A/D mutant in Aurora B depleted cells. Floating mitotic cell were fixed and stained with ACA antibody (red), and DAPI (blue). The quantification of lagging chromosomes (%) from the acquired images (right panel). Bars represent mean ± SEM from three independent experiments; $n_{WT} = 239$, $n_{T236A} = 250$, $n_{T236D} = 242$ mitotic cells. Scale bar, 5 μm. **(c)** Bar graphs represent the fluorescence ratio of Aurora B pT232 against HA staining in Fig 4f. Bars represent mean ± SEM from three independent experiments; $n_{WT} = 28$, $n_{RSF1\ KO} = 34$, $n_{KO+Aurora\ B\ WT} = 46$, $n_{KO+T236A} = 39$, $n_{KO+T236D} = 43$ cells. Source data are provided as a Source Data file.

Supplementary Figure 4

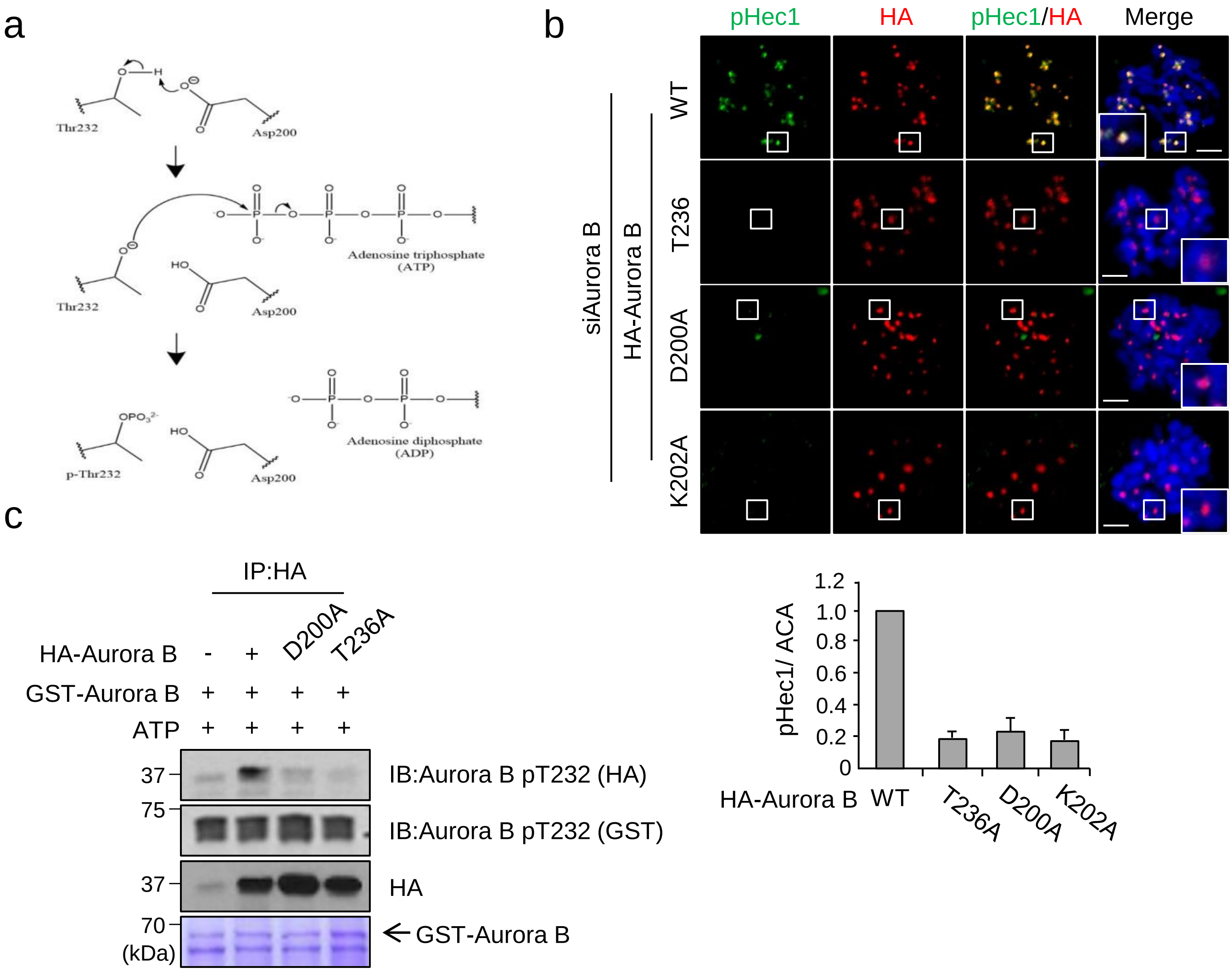

Supplementary Figure 4 The schematic diagram of the general base catalyzed auto-phosphorylation mechanism. **(a)** pThr232 indicates the phosphorylated Thr232. **(b)** HeLa cells transfected with Aurora B D200A or K202A mutants in Aurora B depleted cells. Floating mitotic cell were fixed and stained with anti-Hec1 pS55 antibody (green), anti-HA antibody (red), and DAPI (blue). Bar graphs represent the fluorescence ratio of pHec1 against HA staining (bottom panel). Bars represent mean ± SEM from three independent experiments; n_{WT} = 21, n_{T236A} = 29, n_{D200A} = 32, n_{K202A} = 33 cells. Scale bar, 5 µm. **(c)** The kinase assay with immunoprecipitants of cells expressing Aurora B inactive mutant (D200A, T236A) tagged with HA in the presence of WT Aurora B tagged with GST. Data of c are representative of three independent experiments. Source data are provided as a Source Data file.

Supplementary Figure 5

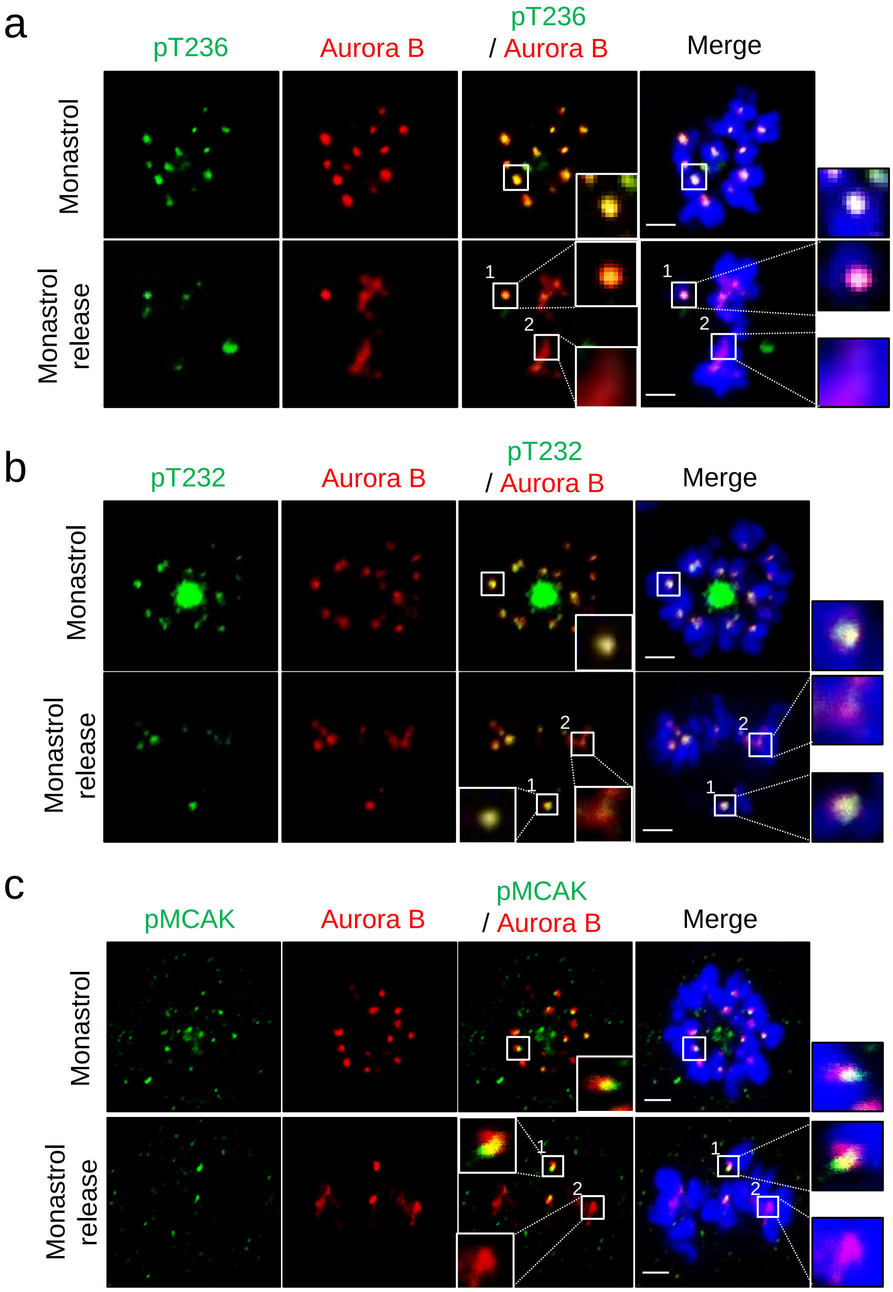

Supplementary Figure 5 Phosphorylation at Thr236 of Aurora B was detected in mis-aligned chromosomes **(a)** HeLa cells treated with monastrol for 4 h and synchronously released to the metaphase for 30 min in the presence of MG132. Floating mitotic cells were stained with anti-Aurora B-pThr236 (green) and Aurora B (red) antibodies and DAPI was used to stain nuclei (blue). Insets show Aurora B-pThr236 staining on mis-aligned (1) vs. aligned (2) kinetochores. Scale bar, 5 μm. **(b)** Floating mitotic cells were stained with anti-Aurora B-pThr232 (green) and Aurora B (red) antibodies. Insets show higher magnification views of the boxed areas. Scale bar, 5 μm. **(c)** Immunofluorescence images acquired using the MCAK pS95 antibodies against Aurora B in monastrol treated HeLa cells. Insets show enlarged mis-aligned or aligned chromosomes from boxed region. Scale bar, 5 μm.

Supplementary Figure 6

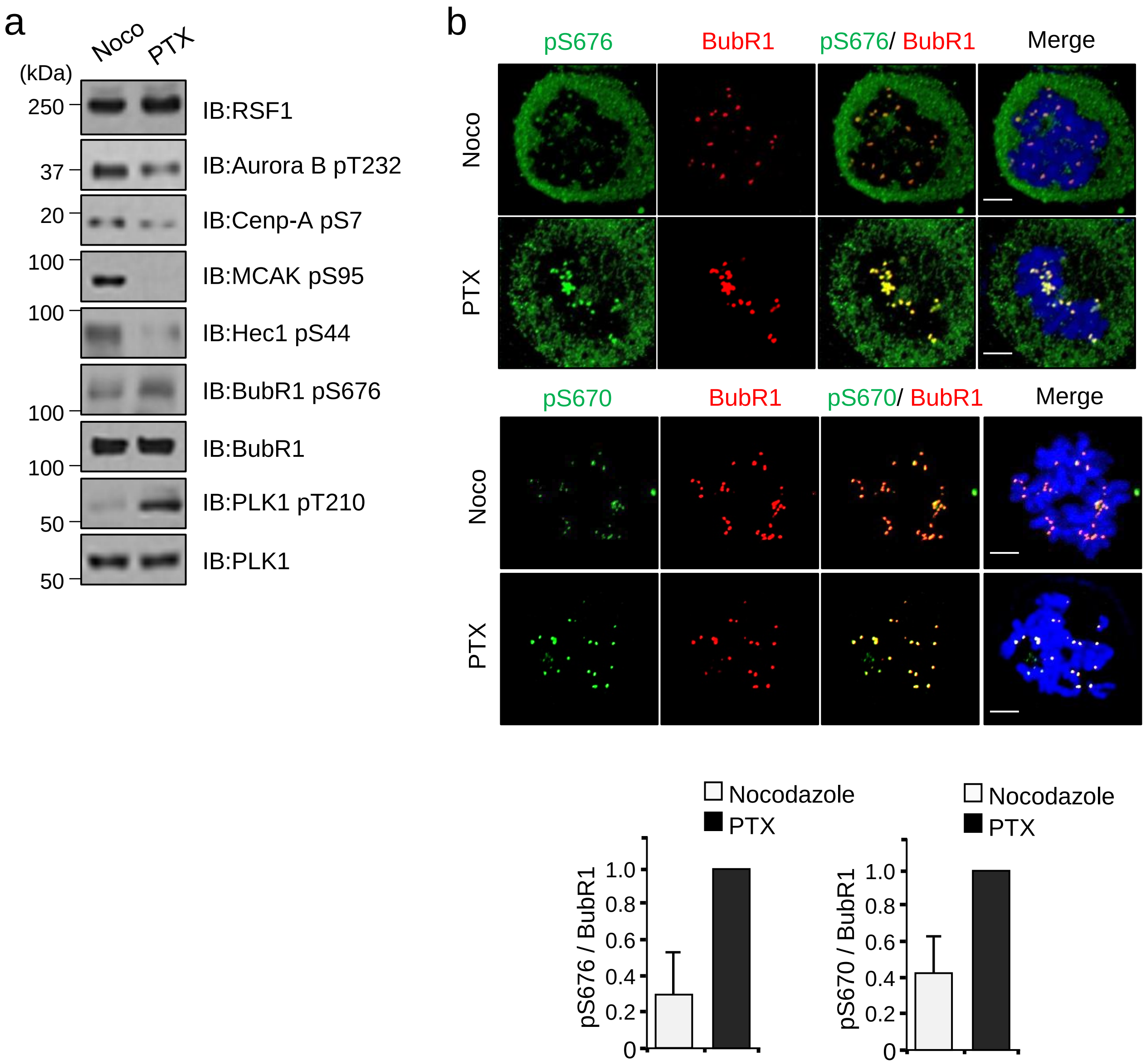

Supplementary Figure 6 Phosphorylation on Ser676 of BubR1 was significantly elevated in paclitaxel-arrested cells **(a)** HeLa cells were treated with nocodazole or paclitaxel for 16 h. Mitotic cells were subjected to Western blot analysis with the indicated antibodies. **(b)** HeLa cells were obtained after nocodazole or paclitaxel treatment for 4 h and subjected to immunostaining. The prometaphase cells were stained with indicated phospho-specific antibodies against BubR1 (green) and total BubR1 (red) were applied for the comparison. Bar graphs represent the fluorescence ratio of BubR1-pT676 ($n_{Noco} = 23$, $n_{PTX} = 25$) or BubR1-pT670 ($n_{Noco} = 22$, $n_{PTX} = 28$) against BubR1 staining (bottom panel). Bars represent mean ± SEM from three independent experiments. Scale bar, 5 µm. Data of a are representative of three independent experiments. Source data are provided as a Source Data file.

Supplementary Figure 7-1

a

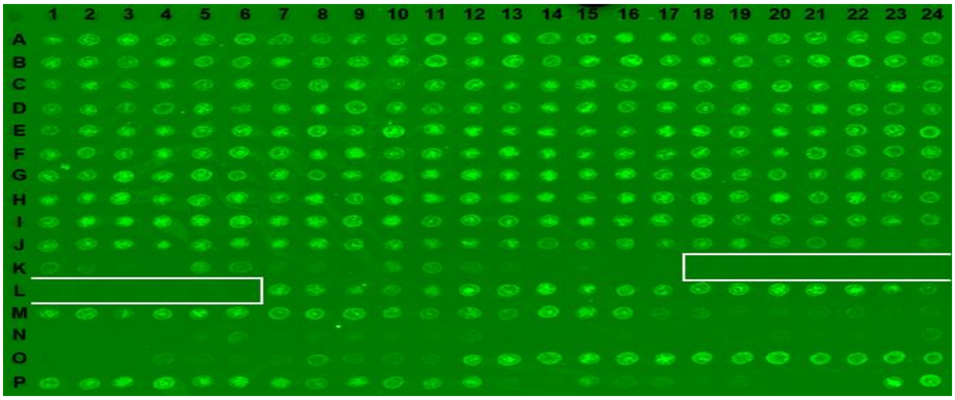

b

Modification number	Peptide location	Peptide sequence	name	Mod1	Mod2	Mod 3	Mod 4	N-terminus
1	A 1	A R T K Q T A R K S T G G K A P R K Q	H3 1-19	unmod				free
220	J 4	A R K S T G G K A P R K Q L A T K A A R	H3 7-26	unmod				acetylated
236	J20	P R K Q L A T K A A R K S A P A T G G	H3 16-35	unmod				acetylated
244	K 4	P R K Q L A T K A A R K pS A P A T G G	H3 16-35	S28P				acetylated
249	K 9	P R K Q L A T K A A Rme2s K pS A P A T G G	H3 16-35	R26me2s	S28P			acetylated
254	K14	P R K Q L A T K A A Rme2a K pS A P A T G G	H3 16-35	R26me2a	S28P			acetylated
258	K18	P R K Q L A T K A A Cit K pS A P A T G G	H3 16-35	R26Citr	S28P			acetylated
259	K19	P R K Q L A T K A A R Kme1 pS A P A T G G	H3 16-35	K27me1	S28P			acetylated
260	K20	P R K Q L A T K A A R Kme2 pS A P A T G G	H3 16-35	K27me2	S28P			acetylated
261	K21	P R K Q L A T K A A R Kme3 pS A P A T G G	H3 16-35	K27me3	S28P			acetylated
262	K22	P R K Q L A T K A A R Kac pS A P A T G G	H3 16-35	K27ac	S28P			acetylated
263	K23	P R K Q L A T K A A Rme2s Kme1 pS A P A T G G	H3 16-35	R26me2s	K27me1	S28P		acetylated
264	K24	P R K Q L A T K A A Rme2s Kme2 pS A P A T G G	H3 16-35	R26me2s	K27me2	S28P		acetylated
265	L 1	P R K Q L A T K A A Rme2s Kme3 pS A P A T G G	H3 16-35	R26me2s	K27me3	S28P		acetylated
266	L 2	P R K Q L A T K A A Rme2s Kac pS A P A T G G	H3 16-35	R26me2s	K27ac	S28P		acetylated
267	L 3	P R K Q L A T K A A Rme2a Kme1 pS A P A T G G	H3 16-35	R26me2a	K27me1	S28P		acetylated
268	L 4	P R K Q L A T K A A Rme2a Kme2 pS A P A T G G	H3 16-35	R26me2a	K27me2	S28P		acetylated
269	L 5	P R K Q L A T K A A Rme2a Kme3 pS A P A T G G	H3 16-35	R26me2a	K27me3	S28P		acetylated
270	L 6	P R K Q L A T K A A Rme2a Kac pS A P A T G G	H3 16-35	R26me2a	K27ac	S28P		acetylated
380	P20	Bio A A N W S H P Q F E K A A	Biotin, control peptide					biotinylated
381	P21	E Q K L I S E E D L A	c-myc tag					free
382	P22	HAc	neg. contol					acetylated
383	P23	K Kme1 Kme2 Kme3 Kac R Rme2s R Rme2a R Cit K Kme1 Kac Kme3 R K	background 0 1					acetylated
384	P24	R Rme2s K Kme1 Kac R Rme2a Kme2 K Kme3 R Kme1 Rme2s K Kac R K	background 0 2					acetylated

c

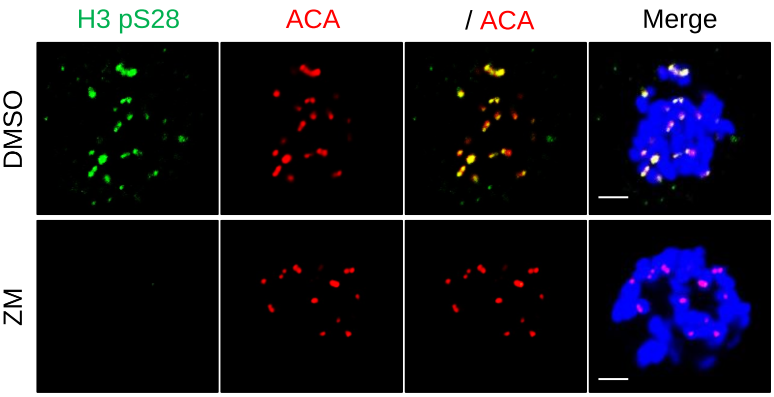

Supplementary Figure 7-2

Supplementary Figure 7 Analysis of the binding specificity of the RSF1 on histone peptide array **(a)** Celluspots peptide arrays spotted on glass slides were provided from Active Motif (Cat. No. 13001). The peptide sequence and PTMs are specified in the additional file 1. Histone peptides containing unique 384 different modification combinations are spotted in the glass slide. Positive interactions of RSF1 protein are visualized as green fluorescence. **(b)** MODified^{TM} Histone Peptide Array and reference grid for histone peptide locations. **(c)** HeLa cells were treated with nocodazole for 4 h and ZM447439 for 30 min in the presence of MG132. The prometaphase cells were stained with anti-Histone H3 pS28 (green) and anti-Aurora B (red) antibodies. Scale bar, 5 μm. Data of c are representative of three independent experiments.

Supplementary Figure 8

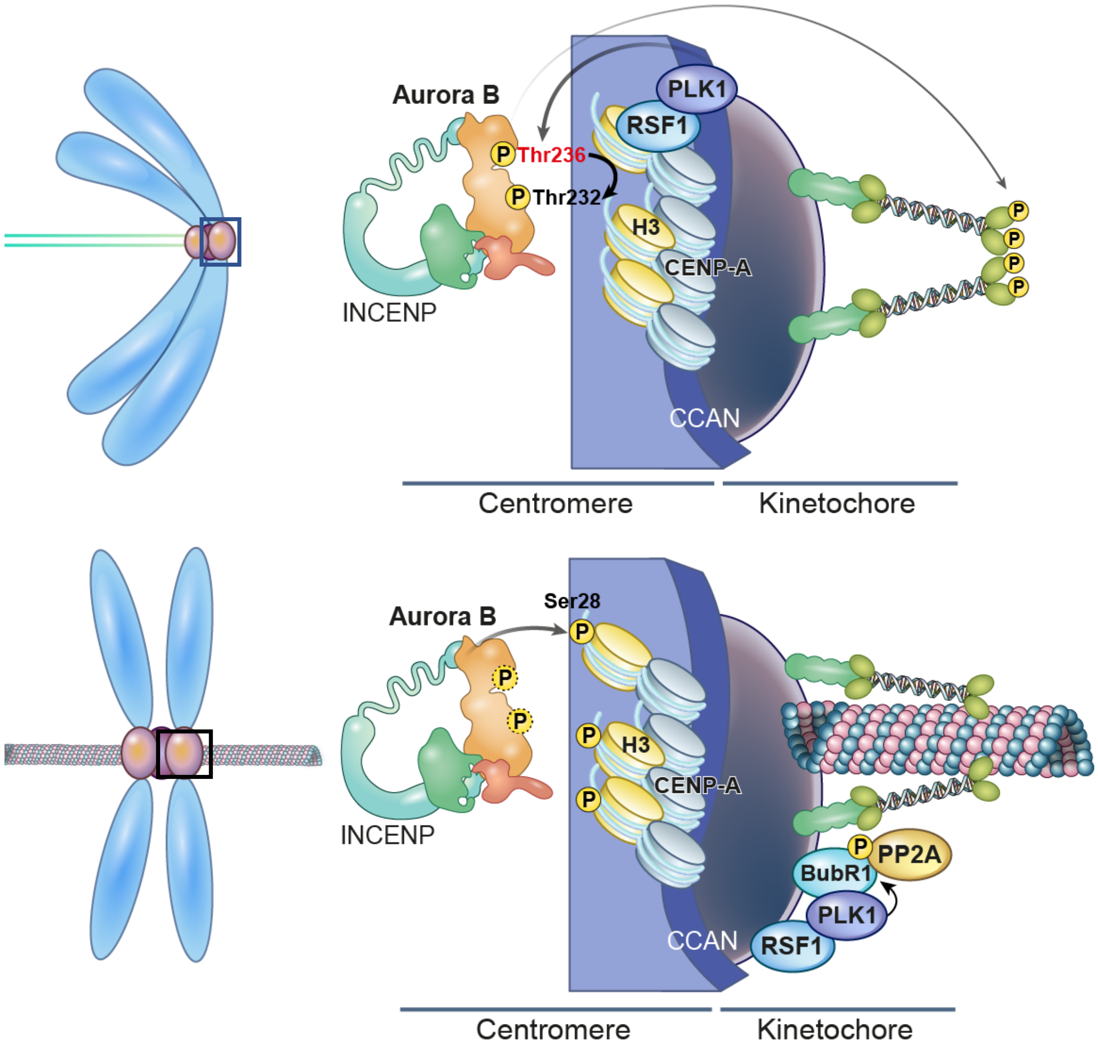

Supplementary Figure 8 A proposed model for the on/off switch on Aurora B by spatiotemporal regulation of the RSF1-PLK1 axis.

Supplementary Table 1

Primers	Sequence (5’-3’)
Mutagenesis forward primer of Aurora B T236A	5’-AAGACAATGTGTGGCGCCCTGGACTACCTGC-3’
Mutagenesis reverse primer of Aurora B T236A	5’- GCAGGTAGTCCAGGGCGCCACACATTGTCTT-3’
Mutagenesis forward primer of Aurora B T236D	5’-GAAGACAATGTGTGGCGACCTGGACTACCTGCC-3’
Mutagenesis reverse primer of Aurora B T236D	5’- GGCAGGTAGTCCAGGTCGCCACACATTGTCTTC-3’
Mutagenesis forward primer of Aurora B D200A	5' GAAGAAGGTGATTCACAGAGCCATAAAGCCAGAAAATCTGC 3'
Mutagenesis reverse primer of Aurora B D200A	5' GCAGATTTTCTGGCTTTATGGCTCTGTGAATCACCTTCTTC 3'
Mutagenesis forward primer of Aurora B K202A	5' GGTGATTCACAGAGACATAGCGCCAGAAAATCTGCTCTTAG 3'
Mutagenesis reverse primer of Aurora B K202A	5' CTAAGAGCAGATTTTCTGGCGCTATGTCTCTGTGAATCACC 3'
Mutagenesis forward primer of Histone H3 S28A	5’ GCGGCTCGGAAGGCCGCTCCGGCCAC 3'
Mutagenesis reverse primer of Histone H3 S28A	5’ GTGGCCGGAGCGGCCTTCCGAGCCGC 3'
Mutagenesis forward primer of Histone H3 S28D	5’ GGCGGCTCGGCCGGACGCTCCGGCCACC 3'
Mutagenesis reverse primer of Histone H3 S28D	5’ GGTGGCCGGAGCGTCCTTCCGAGCCGCC 3'
siRNA oligonucleotide sequence of human Aurora B (targeting 3’UTR)	5’-CGUGUGUUUGUAUGUCUGU-3’
siRNA oligonucleotide sequence of human RSF1 #1	5’-UCGAAACGAGUUGGCUGAGACUCUU-3’
siRNA oligonucleotide sequence of human RSF1 #2	5’-GGAAAAUGUCAAACCCAUU-3’
siRNA oligonucleotide sequence of human SNF2H	5’-AUAGCUCUUCAUCCUCCUCUU-3’

Supplementary Table 1 Primers used in this study.

.

Supplementary Table 2

Antibodies	WB (Dilution)	ICC (Dilution)	Company	Catalog No.
Human anti-ACA		1:2000	Immunovision	HCT-0100
Mouse monoclonal anti-AIM1	1:1000	1:500	BD science	611083
Rabbit polyclonal anti-Aurora B		1:500	Abcam	Ab2254
Rabbit polyclonal anti-Aurora B pT232	1:1000	1:500	Cell Signaling	2914
Rabbit polyclonal anti-Aurora B pT236	1:1000	1:500	Abnova	PAB2635
Mouse monoclonal anti-BubR1	1:1000	1:500	BD science	612505
Rabbit polyclonal anti-BubR1		1:500	Cell Signaling	2186
Rabbit polyclonal anti-BubR1 pS670	1:1000	1:500	N/A	
Rabbit polyclonal anti-BubR1 pS676	1:1000	1:500	N/A	
Mouse monoclonal anti-CENP-A	1:1000	1:500	Abcam	ab13939
Rabbit polyclonal anti-CENP-A		1:500	Cell Signaling	2186
Rabbit polyclonal anti-CENP-A pS7	1:1000	1:500	Cell Signaling	2187
Rabbit polyclonal anti-GAPDH	1:1000		Cell Signaling	2118
Mouse monoclonal anti-GST	1:1000		Santa Cruz	B-14
Mouse monoclonal anti-HA	1:1000	1:500	Santa Cruz	sc-7392
Mouse monoclonal anti-Hec1	1:1000	1:500	abcam	ab3613
Rabbit polyclonal anti-Hec1 pS55	1:1000	1:500	GeneTex	GTX70017
Mouse monoclonal anti-His	1:1000		Santa Cruz	H-3
Rabbit polyclonal anti-INCENP	1:1000	1:500	Abcam	ab36453
Mouse monoclonal anti-M2 Flag	1:1000	1:1000	Sigma	F3165
anti-MCAK	1:1000	1:500	N/A	
Rabbit polyclonal anti-MCAK pS95	1:1000	1:500	abcam	ab74146
Mouse monoclonal anti-Myc	1:1000		Santa Cruz	sc-40
Mouse monoclonal anti-PLK1	1:1000	1:500	Abcam	ab17057
Rabbit polyclonal anti-PLK1		1:500	Santa Cruz	H-152
Mouse monoclonal anti-PLK1 pT210	1:1000	1:500	Abcam	ab39068
Mouse monoclonal anti-PP2A Catalytic α	1:1000		BD science	610555
Mouse monoclonal anti-RSF1		1:500	Upstate	05-727
Rabbit monoclonal anti-RSF1	1:1000	1:500	Abcam	ab109002
Rabbit polyclonal anti-Topoisomerase Ⅱα	1:1000		Santa Cruz	sc-13058
Rabbit monoclonal anti-Histone H3 pS28	1:1000	1:500	Millipore	MABE76
Rabbit polyclonal anti-Histone H3 pS28		1:500	Abcam	Ab5196
Rabbit polyclonal anti-Histone H3	1:1000	1:500	Santa Cruz	Sc-10809
Mouse monoclonal anti-GFP	1:1000		Santa Cruz	Sc-9996
Mouse monoclonal anti-V5	1:1000	1:500	Invitrogen	R960-25
Alexa Fluor 488 goat anti-mouse IgG H&L		1:500	Thermo Fisher Scientific	A-11029
Alexa Fluor 488 goat anti-rabbit IgG H&L		1:500	Thermo Fisher Scientific	A-11034
Alexa Fluor 594 goat anti-mouse IgG H&L		1:500	Thermo Fisher Scientific	A-11032
Alexa Fluor 594 goat anti-rabbit IgG H&L		1:500	Thermo Fisher Scientific	A-11037
Texas Red goat anti-Human IgG H&L			Thermo Fisher Scientific	PA1-28834

Supplementary Table 2 List of antibodies used in this study.
