# Supplementary material for: Spatiotemporal coordination of the RSF1-PLK1-Aurora B cascade establishes mitotic signaling platforms
Source: Nat Commun. 2021 Oct 11;12:5931. doi: 10.1038/s41467-021-26220-z (PMC8505570; doi:10.1038/s41467-021-26220-z)
Supplement: Supplementary file 2 — Reporting Summary [file 41467_2021_26220_MOESM2_ESM.pdf]

nature research

Corresponding author(s): Hyeseong Cho

Last updated by author(s): Jul 27, 2021

Reporting Summary

Nature Research wishes to improve the reproducibility of the work that we publish. This form provides structure for consistency and transparency in reporting. For further information on Nature Research policies, see our Editorial Policies and the Editorial Policy Checklist.

Statistics

For all statistical analyses, confirm that the following items are present in the figure legend, table legend, main text, or Methods section.

n/a	Confirmed	
[ ]	[x]	The exact sample size (n) for each experimental group/condition, given as a discrete number and unit of measurement
[ ]	[x]	A statement on whether measurements were taken from distinct samples or whether the same sample was measured repeatedly
[ ]	[x]	The statistical test(s) used AND whether they are one- or two-sided<br>*Only common tests should be described solely by name; describe more complex techniques in the Methods section.*
[x]	[ ]	A description of all covariates tested
[x]	[ ]	A description of any assumptions or corrections, such as tests of normality and adjustment for multiple comparisons
[ ]	[x]	A full description of the statistical parameters including central tendency (e.g. means) or other basic estimates (e.g. regression coefficient) AND variation (e.g. standard deviation) or associated estimates of uncertainty (e.g. confidence intervals)
[ ]	[x]	For null hypothesis testing, the test statistic (e.g. F, t, r) with confidence intervals, effect sizes, degrees of freedom and P value noted<br>*Give P values as exact values whenever suitable.*
[x]	[ ]	For Bayesian analysis, information on the choice of priors and Markov chain Monte Carlo settings
[x]	[ ]	For hierarchical and complex designs, identification of the appropriate level for tests and full reporting of outcomes
[x]	[ ]	Estimates of effect sizes (e.g. Cohen's d, Pearson's r), indicating how they were calculated

Our web collection on statistics for biologists contains articles on many of the points above.

Software and code

Policy information about availability of computer code

Data collection	Zeiss software: Zen 2011 program<br>Nikon software: NIS elements C program or Ar microscope imaging software
Data analysis	Microsoft Excel and Prism 7 program were used for statistical analysis<br>Image processing and quantification were carried out with ImageJ and Zen program<br>Structural analysis and display were performed using PyMol (https://www.pymol.org/2)<br>Histone Array image was scanned with GenePix 4000B (Axon Istruments, Union City, USA)

For manuscripts utilizing custom algorithms or software that are central to the research but not yet described in published literature, software must be made available to editors and reviewers. We strongly encourage code deposition in a community repository (e.g. GitHub). See the Nature Research guidelines for submitting code & software for further information.

Data

Policy information about availability of data

All manuscripts must include a data availability statement. This statement should provide the following information, where applicable:

- Accession codes, unique identifiers, or web links for publicly available datasets
- A list of figures that have associated raw data
- A description of any restrictions on data availability

We have stated the statistical test in the indicated figure and supplementary figure legends. In addition, we have included the Source Data file in "Data Availability"

Field-specific reporting

Please select the one below that is the best fit for your research. If you are not sure, read the appropriate sections before making your selection.

☒ Life sciences ☐ Behavioural & social sciences ☐ Ecological, evolutionary & environmental sciences

For a reference copy of the document with all sections, see nature.com/documents/nr-reporting-summary-flat.pdf

Life sciences study design

All studies must disclose on these points even when the disclosure is negative.

Sample size	At least 200 mitotic cells were analyzed by mitotic aberration experiments, and at least 40 prometaphse cells were analyzed by immunostaining experiments.
Data exclusions	N/A
Replication	All data were analyzed in three independent experiments
Randomization	N/A
Blinding	Mitotic aberration and immunostaining experiments were blind tested.

Reporting for specific materials, systems and methods

We require information from authors about some types of materials, experimental systems and methods used in many studies. Here, indicate whether each material, system or method listed is relevant to your study. If you are not sure if a list item applies to your research, read the appropriate section before selecting a response.

Materials & experimental systems

n/a	Involved in the study	
☐	☒	Antibodies
☐	☒	Eukaryotic cell lines
☒	☐	Palaeontology and archaeology
☒	☐	Animals and other organisms
☒	☐	Human research participants
☒	☐	Clinical data
☒	☐	Dual use research of concern

Methods

n/a	Involved in the study	
☒	☐	ChIP-seq
☒	☐	Flow cytometry
☒	☐	MRI-based neuroimaging

Antibodies

Antibodies used	Human anti-ACA (Immunovision, HCT-0100); Mouse monoclonal anti-AIM1 (BD science, 611083); Rabbit polyclonal anti-Aurora B (Abcam, Ab2254); Rabbit polyclonal anti-Aurora B pT232 (Cell Signaling, #2914); Rabbit polyclonal anti-Aurora B pT236 (Abnova, PAB2635); Mouse monoclonal anti-BubR1 (BD science, 612505); Rabbit polyclonal anti-BubR1 (Cell Signaling, #2186); Mouse monoclonal anti-CENP-A (Abcam, ab13939); Rabbit polyclonal anti-CENP-A (Cell Signaling, # 2186); Rabbit polyclonal anti-CENP-A pS7 (Cell Signaling, # 2187); Rabbit polyclonal anti-GAPDH (Cell Signaling, # 2118); Mouse monoclonal anti-GST (Santa Cruz, B-14); Mouse monoclonal anti-HA (Santa Cruz, sc-7392); Mouse monoclonal anti-Hec1 (abcam, ab3613); Rabbit polyclonal anti-Hec1 pS55 (GeneTex, GTX70017); Rabbit polyclonal anti-INCENP (Abcam, ab36453); Rabbit polyclonal anti-MCAK pS95 (abcam, ab74146); Mouse monoclonal anti-PLK1 (Abcam, ab17057); Rabbit polyclonal anti-PLK1 (Santa Cruz, H-152); Mouse monoclonal anti-PLK1 pT210 (Abcam, ab39068); Mouse monoclonal anti-PP2A Catalytic α (BD science, 610555); Mouse monoclonal anti-RSF1 (Upstate, 05-727); Rabbit monoclonal anti-RSF1 (Abcam, ab109002); Rabbit monoclonal anti-Histone H3 pS28 (Millipore, MABE76); Rabbit polyclonal anti-Histone H3 pS28 (Abcam, ab5196); Rabbit polyclonal anti-Histone H3 (Santa Cruz, Sc-10809)
Validation	All validation statements were taken from suppliers website.<br>For immunoblotting, the following antibodies were used : Human anti-ACA (Immunovision, 1:1,000); Mouse monoclonal anti-AIM1 (BD science, 1:1,000); Rabbit polyclonal anti-Aurora B pT232 (Cell Signaling, 1:1,000); Rabbit polyclonal anti-Aurora B pT236 (Abnova, 1:1,000); Mouse monoclonal anti-BubR1 (BD science, 1:1,000); Mouse monoclonal anti-CENP-A (Abcam, 1:1,000); Rabbit polyclonal anti-CENP-A pS7 (Cell Signaling, 1:1,000); Rabbit polyclonal anti-GAPDH (Cell Signaling, 1:1,000); Mouse monoclonal anti-GST (Santa Cruz, 1:3,000); Mouse monoclonal anti-HA (Santa Cruz, 1:1,000); Mouse monoclonal anti-Hec1 (abcam, 1:1,000); Rabbit polyclonal anti-Hec1 pS55 (GeneTex, 1:1,000); Rabbit polyclonal anti-INCENP (Abcam, 1:1,000); Rabbit polyclonal anti-MCAK pS95 (abcam, 1:1,000); Mouse monoclonal anti-PLK1 (Abcam, 1:1,000); Rabbit polyclonal anti-PLK1 (Santa Cruz, 1:1,000); Mouse monoclonal anti-PLK1 pT210 (Abcam, 1:1,000); Mouse monoclonal anti-PP2A Catalytic α (BD science, 1:1,000); Rabbit monoclonal anti-RSF1 (Abcam, 1:1,000); Rabbit monoclonal anti-Histone H3 pS28 (Millipore, 1:3,000); Rabbit polyclonal anti-Histone H3 (Santa Cruz, 1:3,000)<br>For immunostaining, the following antibodies were used : Human anti-ACA (Immunovision, 1:1,000); Mouse monoclonal anti-AIM1 (BD science, 1:500); Rabbit polyclonal anti-Aurora B (Abcam, 1:500); Rabbit polyclonal anti-Aurora B pT232 (Cell Signaling, 1:500);

	Rabbit polyclonal anti-Aurora B pT236 (Abnova, 1:500); Mouse monoclonal anti-BubR1 (BD science, 1:500); Rabbit polyclonal anti-BubR1 (Cell Signaling, 1:500); Mouse monoclonal anti-CENP-A (Abcam, 1:500); Rabbit polyclonal anti-CENP-A (Cell Signaling, 1:500); Rabbit polyclonal anti-CENP-A pS7 (Cell Signaling, 1:500); Mouse monoclonal anti-HA (Santa Cruz, 1:500); Mouse monoclonal anti-Hec1 (abcam, 1:500); Rabbit polyclonal anti-Hec1 pS55 (GeneTex, 1:500); Rabbit polyclonal anti-INCENP (Abcam, 1:500); Rabbit polyclonal anti-MCAK pS95 (abcam, 1:500); Mouse monoclonal anti-PLK1 (Abcam, 1:500); Rabbit polyclonal anti-PLK1 (Santa Cruz, 1:500); Mouse monoclonal anti-PLK1 pT210 (Abcam, ab39068); Mouse monoclonal anti-PP2A Catalytic α (BD science, 610555); Mouse monoclonal anti-RSF1 (Upstate, 1:200); Rabbit monoclonal anti-RSF1 (1:200); Rabbit monoclonal anti-Histone H3 pS28 (Millipore, 1:1,000); Rabbit polyclonal anti-Histone H3 pS28 (Abcam, 1:1,000)

Eukaryotic cell lines

Policy information about cell lines

Cell line source(s)	HeLa human cervical cancer cells were used in this study.
Authentication	HeLa cells were originally obtained from the American Type Culture Collection (ATCC, CCl-2)
Mycoplasma contamination	All cell lines were confirmed free of mycoplasma contamination by MycoLuor Mycoplasma Detection Kit (Invitrogen, #M7006)
Commonly misidentified lines (See ICLAC register)	N/A
